# Modelling Human Gut‐Microbiome Interactions in a 3D Bioelectronic Platform

**DOI:** 10.1002/smsc.202300349

**Published:** 2024-04-22

**Authors:** Chrysanthi‐Maria Moysidou, Douglas C. van Niekerk, Verena Stoeger, Charalampos Pitsalidis, Lorraine A. Draper, Aimee M. Withers, Katherine Hughes, Reece McCoy, Rachana Acharya, Colin Hill, Róisín M. Owens

**Affiliations:** ^1^ Department of Chemical Engineering and Biotechnology University of Cambridge Cambridge CB3 0AS UK; ^2^ Department of Physics Khalifa University Abu Dhabi P.O. Box 127788 UAE; ^3^ Healthcare Engineering Innovation Center (HEIC) Khalifa University of Science and Technology Abu Dhabi P.O. Box 127788 UAE; ^4^ APC Microbiome Ireland University College Cork Cork T12 YT20 Ireland; ^5^ Department of Veterinary Medicine University of Cambridge Cambridge CB3 0ES UK

**Keywords:** 3D cell models, barrier integrity, bioelectronics, gut microbiome, host‐microbe interactions, organs‐on‐chips, postbiotics

## Abstract

The role of the gut microbiome in various aspects of health and disease is now a well‐established concept in modern biomedicine. Numerous studies have revealed links between host health and microbial activity, spanning from digestion and metabolism to autoimmune disorders, stress and neuroinflammation. However, the exact mechanisms underlying this complex cross‐talk still remain a mystery. Conventionally, studies examining host‐microbiome interactions rely on animal models, but translation of such findings into human systems is challenging. Bioengineered models represent a highly promisingapproach for tackling such challenges. Here, a bioelectronic platform, the e‐transmembrane, is used to establish a 3D model of human intestine, to study the effects of microbiota on gut barrier integrity. More specifically, how postbiotics and live bacteria impact the morphology and function of the intestinal barrier is evaluated. e‐Transmembrane devices provide a means for in‐line and label‐free continuous monitoring of host‐microbe cross‐talk using electrochemical impedance spectroscopy, revealing distinct patterns that emerge over 24 hours. Microscopy and quantification of molecular biomarkers further validate the differential effects of each bacterial intervention on the host tissue. In addition, a framework to better study and screen drug candidates and potential therapeutic/dietary interventions, such as postbiotics and probiotics, in more physiologically relevant human models is provided.

## Graphical abstract

A novel 3D bioelectronic platform, “e‐Transmembrane”, provides a unique biomimetic niche for the development and dynamic monitoring of an advanced human stratified intestinal model. Exposure of this bioengineered tissue to postbiotic cohorts or live bacteria showcases the unique capabilities of the platform to model gut–microbiota interactions, offering a new and sophisticated way to study such complex systems *in vitro*.

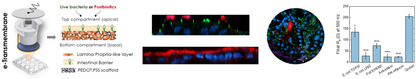

## 1 Introduction

The emergence of gut microbiome as a key regulator of health and disease represents one of the major paradigm shifts in modern biomedicine.^[^ ^1^, ^2^ ^]^ Over the last few decades, the field has experienced tremendous growth, with evidence accumulating on the role of microbiota in food^[^ ^3^, ^4^ ^]^ and drug metabolism,^[^ ^5^ ^]^ in immunity,^[^ ^6^, ^7^ ^]^ in disease onset and progression,^[^ ^8^, ^9^, ^10^ ^]^ as well as in the bidirectional communication between the gut and the brain.^[^ ^11^, ^12^ ^]^ The inner layer of the gut mucosa harbours the intestinal flora, therefore being the first point of contact of dietary components, drug compounds, pathogens/toxins, and other moieties as they pass through the gastrointestinal tract, with the potential to enter the bloodstream via the intestinal epithelium. This, therefore, acts as a selective, dynamic, and functional barrier that controls the absorption and passage of dietary nutrients and potentially harmful intraluminal entities, whilst mediating the interactions between gut microbiota, the resident immune system and enteric nervous system.^[^ ^13^, ^14^ ^]^ Research activity across many fields aims to dissect such mechanisms in various (patho‐)physiological conditions; knowledge that can significantly aid discovery of new therapies and drug targets, including the composition of the gut microbiome per se, as well as improvement of quality of life. In fact, certain microbiota species, with demonstrated effects on the anti‐inflammatory and pro‐inflammatory balance of host responses, have been proposed either as dietary supplements to promote homeostasis or as therapeutic aids in clinical settings.^[^ ^2^, ^15^, ^16^, ^17^, ^18^ ^]^ Over the last years, probiotics, defined as “live microorganisms that, when administered in adequate amounts, confer a health benefit on the host”, have been increasingly used, both by healthy individuals and patients.^[^ ^19^ ^]^ But, recently, various safety concerns have been raised about administering live microbes to critically ill or immunocompromised individuals. Although inactivating microbes via thermal treatments is far from a new concept,^[^ ^20^ ^]^ postbiotics, which refers to “a preparation of inanimate microorganisms and/or their components that confers a health benefit on the host”,^[^ ^19^ ^]^ hold great promise as an alternative and safer option. In fact, postbiotics can interact with the host and influence its microbiome through various pathways,^[^ ^17^, ^19^ ^]^ since they may include microbial cells, their metabolites, cell‐free supernatants, and other bioactive molecules derived from microbial breakdown of carbohydrates, fibres, or proteins.^[^ ^17^, ^21^ ^]^ However, identifying the exact mechanisms of host‐microbiota interplay across space and time (i.e., molecular, cellular, tissue/organ, and organism levels over lifespan), as well as the mechanisms of potential microbiome‐based therapeutics, such as probiotics and postbiotics, has been particularly challenging, not only due to the inherent complexity of this cross‐talk, but mainly due to the lack of appropriate tools.^[^ ^22^, ^23^ ^]^

Germ‐free and gnotobiotic mice are the gold standard model for host‐microbiome studies,^[^ ^24^, ^25^, ^26^ ^]^ which along with novel culture techniques,^[^ ^27^ ^]^ powerful bioinformatic, and next‐generation sequencing technologies,^[^ ^28^ ^]^ keep expanding our understanding of the way microbiota and its alterations affect pathophysiology,^[^ ^8^, ^29^ ^]^ establishing causal links between microbiota profiles and disease phenotypes^[^ ^30^ ^]^ and testing novel treatments,^[^ ^31^, ^32^, ^33^ ^]^ such as faecal microbiome transplantation (FMT).^[^ ^34^, ^35^ ^]^ Although extremely useful, it is now widely accepted that animal models are poor predictors of human systems, due to a series of limitations, including, but not limited to, the inherent difficulties in translating data into clinic, their time‐consuming and labour‐intensive nature and ethical considerations.^[^ ^36^, ^37^, ^38^ ^]^ Recently, in vitro models of human tissues have emerged as highly promising surrogates to animal models. Parallel advancements in and convergence of the fields of stem cell/organoid biology, tissue engineering and organs‐on‐chips (OoCs) have led to the development of a new, more sophisticated generation of tissue models that capture specific hallmarks of human biology.^[^ ^23^, ^39^ ^]^ This is achieved via independent spatiotemporal tuning, control and delivery of key biochemical and biophysical cues, in an advanced, biomimetic microenvironment, featuring key aspects of the tissue‐specific architecture (e.g., villus‐crypt formation, mucus layer). Such bioengineered tissues, interfaced with commensal and/or pathogenic microbiota, were also found to successfully emulate aspects of the human gut‐microbe cross‐talk in vitro.^[^ ^37^, ^40^, ^41^, ^42^ ^]^ For example, intestinal organoids generated from human‐induced pluripotent stem cells (hiPSCs) were successfully used as models of the intestinal epithelium to study the invasion mechanism of *Salmonella enterica serovar Typhimurium*,^[^ ^43^ ^]^ while, in a separate study, the protective role of *Lactobacillus acidophilus* against *S. Typhimurium* infection of the intestinal mucosa was also demonstrated.^[^ ^44^ ^]^ In parallel, OoCs are also exploited for vitro host‐microbiome studies exposing intestinal tissue, derived from cell lines or organoids, to shear‐stress^[^ ^45^, ^46^ ^]^ or mechanical deformation,^[^ ^47^ ^]^ to oxygen gradients,^[^ ^48^, ^49^, ^50^, ^51^ ^]^ and, in some cases, to components of the vascular, connective and immune systems,^[^ ^52^, ^53^, ^54^, ^55^, ^56^ ^]^ as well as to more complex cohorts of the intestinal flora^[^ ^47^, ^48^ ^]^ – all necessary cues for modelling the complex host‐microbiome interplay in vitro. For instance, a human colon mucosal barrier model established in a physiomimetic fluidic platform, namely Gut‐MIcrobiome (GuMI), enabled the long‐term co‐culture and cross‐talk with the super oxygen‐sensitive and hard‐to‐culture *Faecalibacterium prausnitzii,* facilitating the demonstration of its anti‐inflammatory effects.^[^ ^49^ ^]^

Although the utility of this technology for the field of biomedicine is indisputable,^[^ ^39^ ^]^ there still remain a few challenges to address. More specifically, the majority of the OoCs are still quasi‐3D, rely on invasive and/or end‐point characterisation assays and, sometimes, experience scalability/reproducibility issues.^[^ ^57^ ^]^ In addition, most OoC platforms and bioengineered tissues are based on passive materials that lack the ability to respond to stimuli and in‐line monitoring. Inclusion of in‐line sensing capabilities can obviate the need for invasive probes/labels or for time‐consuming and labour‐intensive assays, while at the same time increasing temporal resolution via real‐time evaluation and interrogation of the biological system under study.^[^ ^39^, ^58^ ^]^ Integration of electrical transducers with bioengineered platforms has shown great promise recently.^[^ ^59^ ^]^ A well‐established and popular tool for real‐time monitoring of cells in vitro is electrochemical impedance spectroscopy (EIS), which provides rapid evaluation of parameters such as cell adhesion, proliferation, and differentiation over time. In addition, EIS is also used for obtaining trans‐epithelial/endothelial electrical resistance (TEER), a widely used parameter for the quantification of barrier tissue integrity.^[^ ^60^, ^61^ ^]^ Although, integration of electrodes with OoCs and bioengineered models for impedance spectroscopy is being increasingly explored, in most cases, the electrodes used are ill‐adapted for accurately monitoring complex 3D tissues as they fail to achieve the desired intimate cell‐electrode coupling, and thus are limited in sensitivity and spatiotemporal resolution.^[^ ^62^, ^63^, ^64^ ^]^

An attractive means to overcome this hurdle is presented by organic bioelectronic tools,^[^ ^59^ ^]^ which make use of conducting polymer scaffolds that act both as tissue substrates and as electrodes, seamlessly integrating with the biological system and facilitating accurate signal transduction and recording of its integrity.^[^ ^65^, ^66^, ^67^, ^68^ ^]^ We recently demonstrated the use of such electroactive scaffolds as tissue building blocks‐living electrodes in a 3D bioelectronic transmembrane platform.^[^ ^69^ ^]^ More specifically, we fabricated PEDOT:PSS [poly(3,4‐ethylenedioxythiophene):polystyrene sulfonate] scaffolds to act as separating membranes and electronic units in multi‐well plate platforms for in‐line, non‐invasive monitoring of compartmentalised 3D cultures via EIS.^[^ ^69^ ^]^ However, this was a proof‐of‐concept study, limited to interfacing 3D cell models with the bioelectronic device. In this study, we provide a thorough characterisation of the epithelial tissue established within the e‐Transmembrane devices and we showcase the versatility of the platform for screening the effects of postbiotics and live bacteria on the integrity of the human gut barrier. Specifically, we first present in detail the establishment of the intestinal model in the bioelectronic platform, and we provide a framework/methodology and figures of merit for the evaluation of the tissue integrity by means of EIS monitoring and analysis, in absence and in presence of postbiotics or live bacteria. In particular, we study the host tissue response upon exposure to a simplified human microbiota (SIHUMI)^[^ ^18^ ^]^ postbiotic model, to its pro‐inflammatory and anti‐inflammatory cohorts, as well as to representative live strains of these microbiota. Electrical data, cross‐validated with imaging and molecular biology assays revealed a differential effect of postbiotics and live bacteria, as well as of anti‐inflammatory and pro‐inflammatory bacterial cohorts. Our findings offer important insights into host–microbiota interactions, both at the cellular and at the molecular level, and a new framework for capturing and interrogating certain mechanisms of their complex cross‐talk in vitro. We anticipate that our tissue engineered platforms will serve as valuable tools for further, more mechanistic, microbiome studies, as well as for additional screening of novel treatments/interventions, such as pre‐, pro‐, and post‐biotics.

## 2 Establishment of a 3D Stratified Tissue of the Human Intestinal Barrier, Suitable for Studying Host–Microbiota Interactions

**Figure 1:**
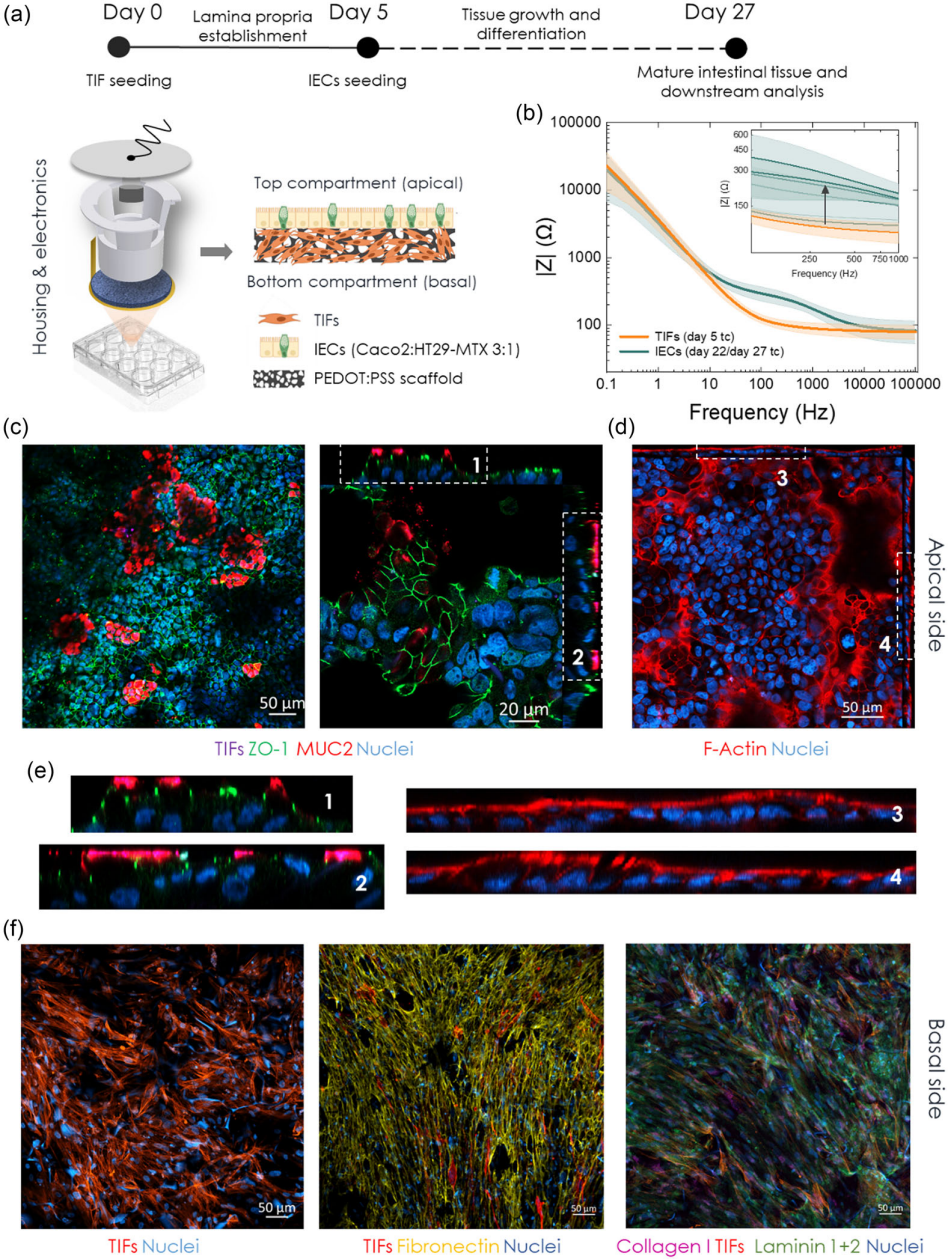
Morphological and functional characterisation of the 3D intestinal model in e‐Transmembrane devices. a) Schematic illustration of the tissue engineering strategy for the establishment of the 3D intestinal barrier model in the e‐Transmembrane bioelectronic platform. The top of the panel indicates the stages and timeline for generating the desired tissue. The schematic underneath illustrates the basic components of the e‐Transmembrane devices and a representation of the tissue morphology within the electroactive scaffolds of the devices: the fibroblast‐derived lamina propria‐like layer covering the bulk compartment of the porous substrate with the IEC‐derived epithelial barrier anchored on top it, covering the apical surface of the scaffolds. b) Bode plot illustrating the impedance magnitude of the devices, hosting the stratified tissue. The orange line corresponds to the mean impedance of the device cultured with fibroblasts, prior to IEC seeding (day 5). The green line corresponds to the mean impedance upon establishment of the intestinal barrier on the fibroblast‐cultured scaffolds, at the end of the experimental period (day 27 of total culture (tc)). The respective colour bands indicate the SD (*N* = 2, *n* = 8). The inset shows the evolution of the impedance magnitude during tissue growth and differentiation, in the mid‐frequency domain. Confocal images of immunofluorescently labelled e‐Transmembrane tissues (day 27). c) Tight junction formation (ZO‐1, in green) and mucin expression (MUC2 in red). d) Brush border formation (filamentous actin in red). e) Magnified *x*/*z* and *y*/*z* orthogonal views of the annotated areas in panels c and d, revealing polarisation of the barrier tissue, and co‐localisation of the relevant biomarkers. f) RFP‐labelled fibroblasts infiltrating the porous network of the scaffolds (left) and depositing ECM proteins – fibronectin (middle, in yellow), collagen I and laminin 1 + 2 (right, in purple and green respectively). All sections were counterstained for nuclei (in blue). Note that black areas in some of the images is an artefact of imaging complex 3D samples in 2D micrographs (also see Figure S2–S4, Supporting Information).

The first step for setting up the bioelectronic tissue of this study involves the fabrication of the e‐Transmembrane devices, a full description of which can be found here.^[^ ^69^ ^]^ Briefly, each e‐Transmembrane insert comprises 1) a PEDOT:PSS‐based porous electroactive scaffold membrane, prepared in disc shape, 400 μm thick and 8 mm wide (culture surface area –28.26 mm^2^), 2) plastic inserts and fittings, facilitating suspension of the e‐Transmembrane scaffold in each well of a 24‐well plate, and thus apical‐basal compartmentalisation of the set‐up, and 3) electrical connections (i.e., gold/working/sensing electrode in contact with the scaffold and Pt mesh/counter/reference electrode on the plate lid) for in situ EIS recordings (**Figure** **1**a, S1a and S1b, Supporting Information). Prior to cell culture experiments, we immerse the fully assembled devices in PBS and run bench‐top electrical recordings to ensure proper performance of each e‐Transmembrane when operated as an electrode for EIS measurements. After this is confirmed, the devices undergo thorough sterilisation, with each insert and accompanying counter electrode being fully immersed in 70% ethanol for ≈2 h, followed by thorough washes with sterile H_2_O and PBS. e‐Transmembranes are then immersed in complete cell growth medium for 2 h, before cell seeding and tissue culture generation. After this stage, we acquire EIS recordings of each device to check their electrical properties and performance prior to interfacing with cells (Figure S1c, Supporting Information, also see^[^ ^69^ ^]^).

To model the human intestinal epithelial barrier, we employ a two‐stage tissue engineering strategy, adapted from our previous work^[^ ^68^, ^69^ ^]^ (Figure 1a). First, human telomerase immortalised fibroblasts, (TIFs; RFP LifeAct‐transfected) are seeded on the apical surface of the e‐Transmembrane scaffolds and incubated for ≈1.5–2 h before immersion in TIF‐complete cell growth medium (day 0 of total culture (tc)). This 3D culture system is then maintained for 4 days to allow TIFs to infiltrate the bulk of the scaffolds and deposit extracellular matrix proteins (ECM). As a result, on day 5 tc, there is already a connective tissue‐like layer formed on the electroactive substrate, which acts as an anchoring point and a guide for the subsequently seeded intestinal epithelial cells (i.e., 3:1 Caco‐2/HT29‐MTX cells; IECs), blocking their access to the bulk of the porous substrate while enhancing their adherence on its apical surface. This forces IECs to then form the desired epithelial monolayer, supporting it throughout the experimental period. After further maintenance of the tri‐culture system for another three weeks, it is expected that a stratified tissue is formed in each e‐Transmembrane, emulating the human intestinal barrier, underlined by the lamina propria‐like layer found in the native tissue.

Throughout the almost one‐month long tissue formation in the e‐Transmembranes, we operate the devices as electrodes, (as described in ref. [69]), to assess the response of the conducting scaffold to cell adherence, proliferation, and differentiation into a barrier tissue. Figure 1b illustrates the evolution of EIS output of the e‐Transmembranes during tissue formation, which is in agreement with previous observations. On day 5, which corresponds to TIF‐cultivated scaffolds, the impedance profile of the scaffolds exhibits similar features as the cell‐free scaffolds (Figure S1c, Supporting Information): a flat ohmic line, parallel to the x‐axis, at the high‐ to mid‐frequency region – a measure of the electrolyte resistance – while the low‐frequency region is dominated by the counter electrode capacitance. Upon formation of the barrier tissue, we observe a distinct increase of the overall impedance magnitude in the mid‐frequency range (inset of Figure 1b), with the corresponding curve shape in the Phase plots being indicative of the presence of a barrier tissue in the device and the characteristic time constants (Figure S1d, Supporting Information), which is in line with studies in literature based on impedance spectroscopy of cell‐cultures.^[^ ^60^, ^61^, ^70^ ^]^ This impedance signature indicates that the tissue model has developed a component which interacts with ionic flow in such a way that it offers two parallel current pathways, one resistive and the other capacitive, reflecting the paracellular and transcellular pathways of the barrier tissue, respectively.

To validate our electrical recordings and the tissue engineering strategy, we performed immunofluorescence microscopy at the end of the experiment (i.e., day 27 of tc/day 22 of IEC culture). After fixing the cells within the e‐Transmembrane platform, we then disassemble the devices, remove the electroactive substrates, and stain the tissue for key intestinal barrier biomarkers. Fibroblasts are RFP‐transfected, simplifying our staining protocol. Figure 1c,d reveal the formation of a continuous, homogenous intestinal monolayer on the e‐Transmembrane scaffolds, with extensive expression of the tight junction (TJ) protein Zonula Occludens‐1 (ZO‐1), mucin 2 protein (MUC‐2), and filamentous actin (F‐actin). Z‐stacked confocal images and the reconstructed *x*/*z*, *y*/*z* ortho‐views (Figure 1c, right, 1d, 1e (magnified ortho‐views of annotated 1c and 1 d panels), Figure S2 and S3, Supporting Information) illustrate the cell/tissue organisation, polarisation of the monolayer and localisation of these biomarkers after the ≈one‐month experiment. The characteristic chicken‐wire/mosaic‐like ZO‐1 pattern is revealed, indicative of an epithelial barrier in which ZO‐1 is internally bracing adjacent cells, in a ribbon‐like distribution to seal and stabilise the paracellular pathway. In addition, ZO‐1 is found in the apical domain of the columnar epithelial cells of the monolayer (Figure 1e), similar to in vivo barriers,^[^ ^71^, ^72^ ^]^ and co‐localised with MUC2 domains, validating the apical‐basal compartmentalisation of the tissue. This was further confirmed with F‐actin staining (Figure 1d), which additionally revealed the formation of a typical apical brush border (Figure 1e). Importantly, the monolayers in the ortho‐views (Figure 1e) do not appear as straight bands, but rather as flowing ribbons. This was not unexpected given the 3D microenvironment and the relatively rough surface of the electroactive, porous substrates. In addition, this renders capture of all tissue features in one, 2D plane challenging, resulting in black areas in the 2D micrographs. However, this was bypassed by z‐stack confocal microscopy assays, allowing for reconstruction of 3D renderings that revealed a continuous intestinal barrier tissue on the apical surface of the TIF‐cultured e‐Transmembrane scaffolds (Figure S4, Supporting Information). Moreover, we also imaged the bulk of the scaffolds to verify the establishment and maintenance of lamina propria element/layer of our tissue engineering strategy. In addition to imaging fibroblasts by taking advantage of the intrinsic RFP label, using immunofluorescence we labelled key ECM proteins, again at the end of the ≈one‐month culture. As postulated, a fibrous‐like network of fibroblasts was found in the bulk porous network of the electroactive substrates, with cells coating the surface of the scaffolds, but also occupying the pore cavities and depositing copious amounts of fibronectin, collagen I and laminin (Figure 1f and S5, Supporting Information). Finally, to further validate our tissue engineering strategy, we performed histological analysis of thin cross‐sections of the e‐Transmembrane scaffolds. This revealed the expected tissue stratification, with localisation of the intestinal cell‐specific (i.e., epithelial cytokeratins, PAS, Alcian blue) and fibroblast‐specific (i.e., vimentin) biomarkers in the apical and bulk domains of the scaffolds respectively (Figure S6, Supporting Information). The stratification of the tissue and localisation of the epithelial and stromal layers were also confirmed by scanning electron microscopy (SEM) (Figure S7, Supporting Information), revealing a continuous and homogenous epithelial layer on the apical surface of the scaffolds, underlined by a fibroblast‐derived stromal layer established in the bulk of the scaffolds.

## 3 Emulating Host‐Microbe Interactions in the e‐Transmembrane Gut Barrier Tissue Models

Previously, we showed that the e‐Trasmembranes can detect real‐time breaches and recovery of epithelial barriers, rapidly, in a non‐invasive, label‐free manner, and with remarkable sensitivity.^[^ ^69^ ^]^ Taking advantage of such in‐line sensing features, we herein use e‐Transmembrane 3D intestinal tissues to emulate and monitor in real‐time the interactions of gut microbiome bacteria with the host tissue, with a particular focus on their effects on gut barrier integrity at a cellular and a molecular level. In addition, we seek to understand whether such effects are different when the host tissue is exposed to single or simplified, or to inanimate or live bacterial consortia, with distinct properties.

### 3.1 Effects of Postbiotics on Intestinal Barrier Integrity

The first set of questions aimed to examine changes in the intestinal barrier integrity when exposed to anti‐inflammatory and pro‐inflammatory bacteria, as well to a combination of those cohorts. To this end, we used six members of a postbiotic preparation of a well‐characterised simplified human microbiome (SIHUMI) model: *Escherichia coli* LF82*, Ruminococcus gnavus* ATCC 29 149*, Enterococcus faecalis* OG1RF*, Bifidobacterium longum* ATCC 15 707*, Faecalibacterium prausnitzii* A2‐165*, Phocaeicola vulgatus* DSM 1447 *(*formerly *Bacteroides vulgatus).* We chose this cohort because, although of reduced complexity, it represents a robust, reproducible, and physiologically relevant in vitro microbiota model^[^ ^73^ ^]^ that contains bacterial strains of key genres of the human intestinal flora, with a broad range of properties (e.g., obligate anaerobes, probiotics) and known for their implication in various (patho‐)physiological conditions. We exposed our tissue to a single inoculum of this microbial community in order to address our questions.

The use of the SIHUMI cohort and sub‐cohorts allowed us to address our questions in different scenaria/applications. As mentioned earlier, the use of postbiotics as microbiota‐modulating dietary/pharmacological interventions has gained a lot of interest over the past few years.^[^ ^19^ ^]^ However, translation of such products in the market and in clinical settings has been delayed due to lack of robust preclinical and clinical models that could provide strong evidence on the specific mechanisms of action of different postbiotic consortia and their effects on host health, and thus allow the development of safe and efficacious postbiotics. Here, using our e‐Transmembrane intestinal tissue models, we were able to investigate how postbiotic commensal consortia with distinct properties interact with the host tissue, providing evidence of their effects on the intestinal epithelium morphology, function, and integrity.

**Figure 2:**
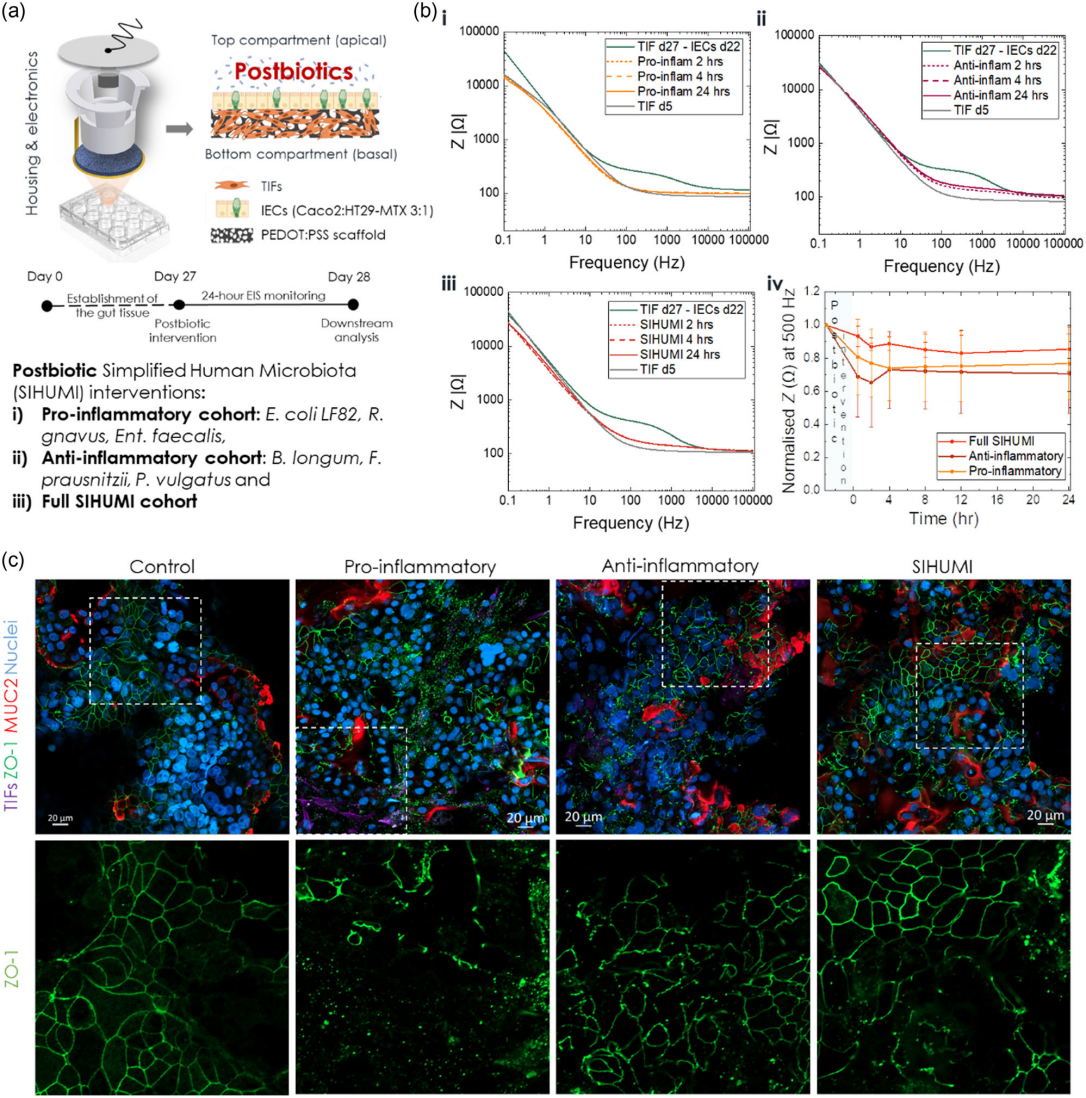
Effect of postbiotics on the gut barrier integrity. a) Schematic illustration of the experimental design and types of postbiotic interventions in the e‐Transmembrane gut tissues. b) Bode plots illustrating the evolution of the impedance magnitude of one representative e‐Transmembrane during the 24‐hour exposure to each postbiotic cohort (i: pro‐inflammatory, ii: anti‐inflammatory, iii: full SIHUMI). Panel iv depicts the impedance magnitude cross‐section at 500 Hz during the intervention, normalised to the baseline impedance magnitude (prior to postbiotic addition). Each data point represents the mean ± SD (*N* = 2, *n* ≥ 3). c) Confocal images of immunofluorescently labelled tissues for barrier and brush border biomarkers (ZO‐1 in green, MUC2 in red, counterstained for nuclei in blue) before and after exposure to each postbiotic intervention. Insets in panels in the top row correspond to magnified areas of each image in the bottom row, illustrating ZO‐1 tight junction network.

To prepare the postbiotic intervention, the strains that constitute the SIHUMI cohort were cultured in Wilkins‐Chalgren Anaerobe Broth (Oxoid) at 37 °C in Hungate tubes to maintain anaerobicity. After overnight growth, bacterial numbers were assessed (see **Table** **1**) and then each culture (i.e., 6 individual strains and the full cohort) were thermally inactivated (i.e., autoclaved at 121 °C for 15 min). Prior to the interventions, respective strains for the pro‐inflammatory and anti‐inflammatory cohorts were mixed in the ratios they typically fall into in the full SIHUMI cohort.^[^ ^74^ ^]^ On day 22 of IEC culture/day 27 tc, we remove media from the apical compartment of host‐tissue free or gut tissue e‐Transmembranes and expose them to ‘intervention media’ (i.e., tri‐culture composition media but with reduced amounts of FBS and antibiotics as described here^[^ ^75^ ^]^). Host‐tissue free e‐Transmembranes act here as controls to check whether the addition of postbiotic inocula will affect the EIS measurements. After ≈one hour of incubation, we take an EIS measurement that represents the baseline for each intervention. Then media are removed, and dedicated devices are inoculated with the respective intervention (**Figure** **2**a). The host‐postbiotic cocultures are monitored continuously for 24 h post‐inoculation via EIS recordings at pre‐determined time points (i.e., 30 min, 2, 4, 8, 12, 24 h).

**Table 1:** Concentration (CFU/ml) of each strain when grown in a steady state fermentation

*F. prausnitzii*	*R. gnavus*	*E.coli LF82*	*E. faecalis*	*P. vulgatus*	*B. longum*
1.6 × 10^−2^	8.0 × 10^5^	1.7 × 10^7^	1.8 × 10^7^	1.4 × 10^8^	2.6 × 10^8^

Figure 2b illustrates the effects of the interventions on the intestinal barrier integrity of e‐Transmembranes tissue as recorded via EIS. In all cases, from the first hours post‐inoculation, there is a decrease of the impedance magnitude and changes in the shape of the curve, particularly in the mid‐frequency regime, indicative of a decreasing resistance of the paracellular pathway and thus a disruption in barrier function. This shift of the impedance profile is more dramatic in the case of the proinflammatory cohort (Figure 2b,i), with the whole curve shifting back to the profile of TIF‐cultured scaffolds on day 5 tc, prior to seeding intestinal cells, showing no signs of recovery, but rather plateauing at this level. In contrast, when the e‐Transmembrane tissues are exposed to the anti‐inflammatory cohort, we observed a more conservative drop towards lower impedance values, but this drop was limited to the mid‐frequency regime and the barrier appeared to try to recover its integrity after 24 h, although not completely returning to baseline impedance levels during that time‐frame (Figure 2b,ii). The impedance profile when the full SIHUMI cohort is inoculated appears to be a combination of the pro‐inflammatory and anti‐inflammatory EIS profiles; again there is a decrease of the overall impedance magnitude, more prominent in the mid‐frequency regime, but to a greater degree than the anti‐inflammatory cohort and not as dramatic as in the proinflammatory cohort, while the impedance converges to the level it initially dropped to, with no signs of recovery (Figure 2b,iii). Note that Bode plots in Figure 2b were obtained from representative e‐Transmembrane gut tissues exposed to each intervention. However, the response trend was consistent across all devices of respective interventions, as summarised in the cross‐section of the impedance magnitude at 500 Hz (Figure 2b,iv), which consistently lies within the frequency regime of the impedance spectrum that is dominated by paracellular resistance, and as a result, highly correlated with the function and integrity of the barrier paracellular pathway.

To cross‐validate the electrical recordings and to visualise the effects of the postbiotic interventions, we employed confocal microscopy, as before. As seen in Figure 2c, our findings aligned with our electrical recordings. In all cases, staining the barrier tissue for ZO‐1 and MUC2 revealed a disturbed epithelial layer, compared to tissues that were not exposed (please note that these e‐Transmembrane tissues were not exposed to any intervention and are used in these comparisons as controls). The ZO‐1 pattern appears intact in some areas, but also faint and disarranged in others, with these effects being more dramatic in the case of the pro‐inflammatory postbiotic inocula, which was not an unexpected outcome, given the properties of the bacterial members of this intervention. What was surprising, however, was the fact that the underlying TIF‐tissue layer can be seen through “holes”/breached spots in the intestinal tissue (see annotated inset in Figure 2c, S7 and S8, Supporting Information), which is not the case when the monolayer is intact, prior to the interventions (control in Figure 1c and 2c). This was not observed in the rest of the interventions, where the TIF layer could not be seen in areas that exhibit a breached ZO‐1 network. In addition, such breaches are not as extensive in the anti‐inflammatory and SIHUMI interventions (see annotated areas in Figure 2c, S9 and S10, Supporting Information). Moreover, MUC 2 domains appear relatively expanded/swollen, compared to the control tissue (see Figure 1c,e, S10 and S11, Supporting Information). Similar observations were made with F‐actin staining, with the monolayers exhibiting disturbance and re‐arrangement in some locations, while in others the rich in microvilli brush border appears dense and intact (Figure S9–S11, Supporting Information). Of note, in the case of pro‐inflammatory intervention, TIF cells are also revealed in breached areas of the F‐actin stained monolayer. Despite the difficulty in distinguishing between IECs and TIFs in this case, due to the overlap of the RFP reporter in TIFs and the F‐actin label staining, the shape and morphology of cells in breached areas points towards this conclusion (Figure S9–S11, Supporting Information).

### 3.2 Effects of Live Bacteria on Intestinal Barrier Integrity

**Figure 3:**
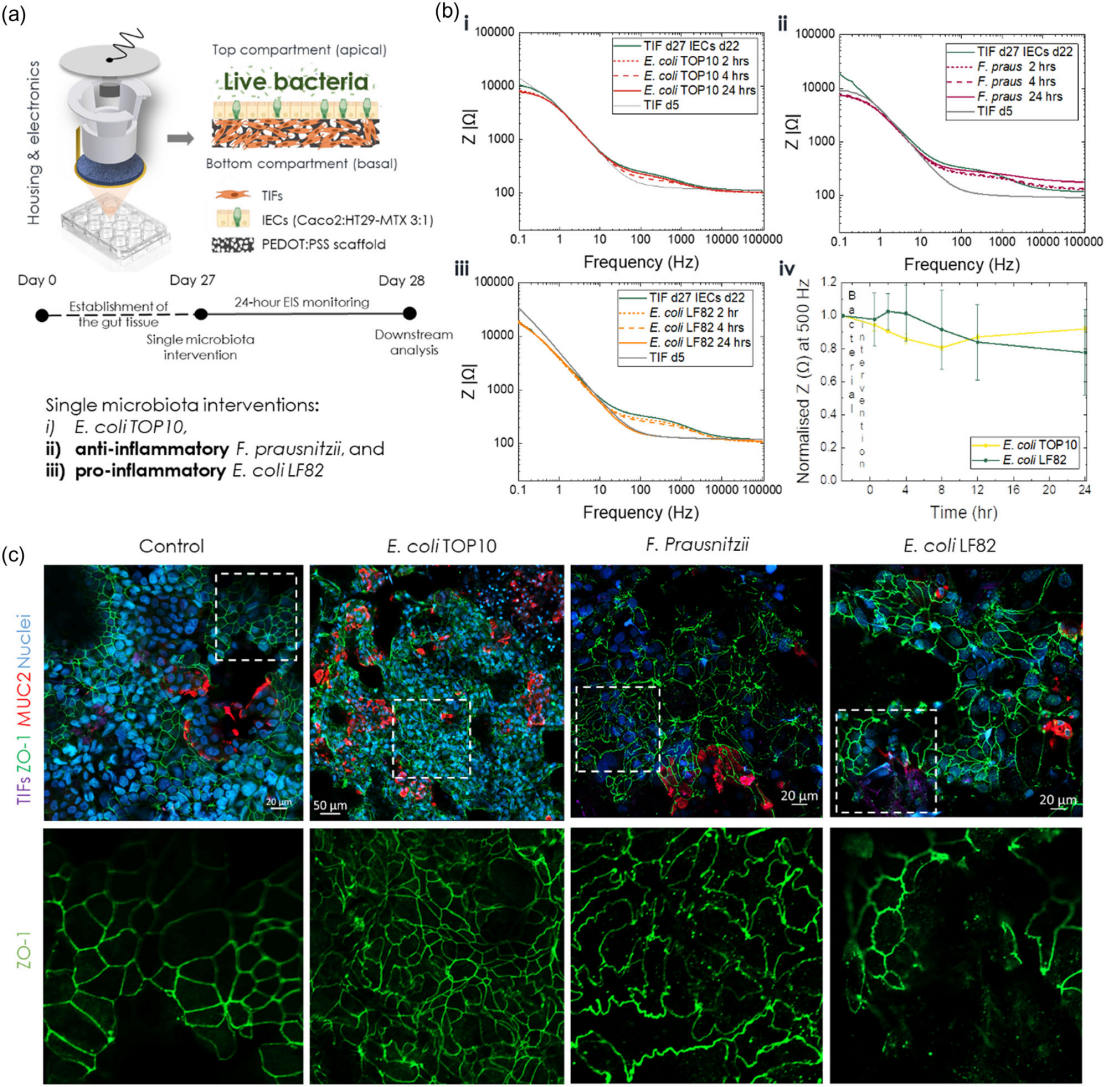
Effect of live bacteria on the gut barrier integrity. a) Schematic illustration of the experimental design and types of live bacteria interventions in the e‐Transmembrane gut tissues. b) Bode plots illustrating the evolution of the impedance magnitude of one representative e‐Transmembrane during the 24‐hour exposure to each bacterial strain (i: *E. coli* TOP10, ii: *F. Prausnitzii*, iii: *E. coli* LF82). Panel iv depicts the impedance magnitude cross‐section at 500 Hz during the intervention, normalised to the baseline impedance magnitude (prior to bacterial addition). Each data point represents the mean ± SD (*N* = 2, *n* ≥ 3). c) Confocal images of immunofluorescently labelled tissues for barrier and brush border biomarkers (ZO‐1, MUC2, counterstained for nuclei) before and after exposure to each bacterial strain. Insets in panels in the top row correspond to magnified areas of each image in the bottom row, illustrating ZO‐1 tight junction network.

Next, we shifted our focus to the interactions of the host tissue with live bacteria. To this end, we chose one simple bacterial strain of commensal origin, *E. coli* TOP10, transformed to express GFP, and two strains that are members of the SIHUMI cohort: i) the obligate anaerobe *F. prausnitzii,* a common strain in the intestinal flora of healthy adults, with well‐known anti‐inflammatory properties,^[^ ^49^, ^76^, ^77^ ^]^ and ii) the facultative anaerobe adherent/invasive *E. coli* LF82, which is associated with inflammatory gastrointestinal disorders, such as IBD and IBS,^[^ ^78^, ^79^, ^80^ ^]^ (**Figure** **3**a).

We have previously demonstrated the establishment of a stable, short‐term co‐culture of *E. coli* TOP10 GFP with the IEC‐derived monolayer in a hydrogel‐based 3D model of the vertebrate gut.^[^ ^75^ ^]^ Here, we employ a similar approach for interfacing the e‐Transmembrane intestinal tissue with all three bacterial strains, modifying the protocol to accommodate growth and maintenance of *F. prausnitzii.* Briefly, each bacterial strain grows in its optimum culture conditions until exponential phase, when the desired number of cells is retrieved (see Methods). Prior to co‐culture, on day 22 of IEC culture /day 27 tc, each bacterial cell suspension is washed twice with PBS and resuspended in intervention media (i.e., modified tri‐culture media as described here^[^ ^75^ ^]^) for a stable host‐microbe coculture. In the case of *F. prausnitzii,* this medium was deoxygenated prior to use. Before adding bacterial inocula, apical e‐Transmembrane medium is removed, and the tissue is gently rinsed with the aforementioned modified growth medium. Upon inoculation, co‐cultures were again maintained for 24 h, during which EIS measurements were obtained at dedicated time points. It is important to note that devices inoculated with *F. prausnitzii* were maintained in an anaerobic chamber, while measurements were taken in an anaerobic glove box, both set at 0.1% O_2_, 37 °C, with the former also maintaining 5% CO_2_.

The effects of each live bacterial intervention on the intestinal barrier integrity of e‐Transmembranes, as recorded via EIS, are illustrated in Figure 3b. In contrast to postbiotic interventions, after the first few hours of interaction the impedance magnitude drops gradually in all cases, while exhibiting signs of recovery at later stages, with the exception of *E. coli* LF82. More specifically, the host tissue interfaced with *E. coli* TOP10 exhibits a slight decrease of the impedance magnitude in the mid‐frequency domain at 2 h, which drops further at 4 h, but it reverts, almost, to its initial levels after 24 h (Figure 3b,i). A similar trend is observed in the case of the *F. prausnitzii*, however the whole impedance curve at 24‐hours is shifted towards higher values, with the shift being more dramatic in the mid‐ and high‐frequency domains (Figure 3b,ii). It is likely that the hypoxic environment affected the EIS measurements, reflected in this trend of the impedance curve. Although slight variations in the overall impedance profile of the host tissue‐free/blank e‐Transmembrane exposed to *F. prausnitzii* (Figure S12, Supporting Information) could support this hypothesis, the small increase observed at 24‐hours post‐inoculation extends across the measured frequency range. We therefore checked whether the anaerobes survived after the co‐culture experiment by collecting apical supernatant from the devices and maintained them in an anaerobic chamber for several days, regularly checking for signs of growth. Contrary to the other two bacterial strains which survived the 24‐hour interaction with the host, we could not detect any colonies of *F. prausnitzii* (Figure S13, Supporting Information), indicating that the co‐culture conditions were not appropriate. Although we used high‐tech equipment, designed for experiments that require specific atmosphere conditions, and we deoxygenated the inoculation media for 48 h before the host‐microbe co‐culture, we postulate that the oxygen concentration might have been higher than the optimal for *F. prausnitzii* survival (e.g., despite the proximity of glove box to the anaerobic chamber (see M&M) the few seconds that would take to transfer the samples to the glove box for the measurement may have compromised oxygen concentration). Additionally, the presence of FBS (2%) and bacteriostatic concentration of antibiotics (0.5%) in the inoculation media may have also affected *F. prausnitzii* survival during co‐culture with the host tissue. Therefore, the observed increase in the impedance should not be solely interpreted as an *F‐prausnitzii* mediated reinforcement of the barrier, but rather as a potential recovery of the host tissue upon inactivation/death of bacteria. Further experiments are required to optimise the co‐culture protocol and microenvironment parameters for testing e‐Transmembrane tissue with anaerobic bacteria (e.g., optimisation of the experimental setup and the composition of inoculation media). Furthermore, the *E. coli* LF82 intervention resulted in a drop of the impedance magnitude, reaching the levels of TIF‐cultivated scaffolds at day 5, similar to the pro‐inflammatory postbiotic case. The decreasing trend of the impedance, however, was not the same, with the drop here being more limited during the first hours post‐inoculation (Figure 3b,iii), compared to the rapid, dramatic shift observed in the postbiotic case. Note that, as before, Figure 3b illustrates Bode plots of samples representative of each intervention, while the trends observed are consistent across experiments and summarised with a cross‐section of the impedance magnitude at 500 Hz (Figure 3b,iv).

We then turned to confocal microscopy to optically evaluate changes in the morphology and structure of the e‐Transmembrane barrier tissue upon live bacteria interventions, following the same immunofluorescent staining assays as before. In the case of *E. coli* TOP10, the ZO‐1 tight junction network and mucus domains appear minimally disturbed, while the F‐actin staining revealed a few areas where individual cells appear rounded, slightly detached from neighbouring cells (Figure 3c, see annotated areas and Figure S14 and S15, Supporting Information). These observations align with our in‐line monitoring findings, suggesting dynamic rearrangement of the barrier towards the end of the experimental duration, as well as with similar studies in literature, including previous applications by our group.^[^ ^75^ ^]^ Similarly, the tight junction network of host tissue exposed to *F. prausnitzii* exhibits signs of recovery and resealing of the paracellular pathway. However, a closer look reveals a faint patterning of ZO‐1, with some cells being detached from adjacent ones (Figure 3c, see annotated areas and Figure S14 and S16, Supporting Information). In addition, fewer mucus domains were detected, and filamentous actin network and brush border appear disturbed/disorganised/inhomogeneous (Figure S16, Supporting Information). Such effects could be attributed to exposure of the barrier tissue to very low levels of oxygen, as mentioned before, or to a combination of this microenvironment with bacterial death during the co‐culture. Finally, immunofluorescent staining of e‐Transmembrane barriers exposed to *E. coli* LF82 revealed breaches of the ZO‐1 tight junction network, which may retain a degree of intactness is some locations, but it appears severely damaged in others. In fact, the underlying TIF‐layer can be clearly seen in such affected areas, similarly to the findings of the pro‐inflammatory postbiotic intervention and aligning with the EIS recordings. We detected fewer mucus domains in this intervention too, while the filamentous actin in both cytoskeletal regions and in the apical brush border demonstrates – similar to ZO‐1 staining – both intact and breached regions in the epithelium (Figure S14 and S17, Supporting Information).

### 3.3 Evaluation of Host‐Microbe Interactions in the e‐Transmembranes

Although EIS recordings and confocal microscopy offered valuable input on how postbiotic and live bacterial interventions affect the structural and functional properties of the gut barrier tissue, examination of the raw impedance spectra is merely qualitative. Furthermore, raw impedance data is by nature an aggregate of the various electrochemical phenomena (See Figure S1d, Supporting Information) of which the electrochemical cell is comprised; this superposition confounds the data and information pertaining specifically to the biological components of the total system needs to be deconvoluted. The electrochemical phenomena present within a system such as this are primarily of a resistive (frequency independent) or capacitive nature. The inverse proportionality of a capacitive impedance results in the contribution of said element to the measured impedance (such as the contribution of the electrochemical processes occurring at the electrode‐electrolyte interfaces) tending asymptotically to zero with frequency. It is therefore possible to design a bioimpedance sensor such that the region in frequency wherein the biological impedance components are dominant are well separated from the contribution of the abiotic elements of the system. In this ideal case, the non‐specificity inherent to EIS, mentioned above, would be mitigated and the raw impedance (such as that shown in Figure 2b,iv), at an appropriate frequency, would be correlated exclusively to the biological state. While the e‐transmembrane design is rationalized in this way, by maximizing the counter and working electrode surface areas (and thus capacitances), it is, in practice, challenging to truly deconvolute the biological and abiotic impedance contributions by design alone. Separation of the constituent sources of variance in EIS data is most commonly achieved by way of equivalent circuit fitting^[^ ^60^, ^81^ ^]^ and corroborated by auxiliary measurements of biological state. In order to acquire a better understanding of host‐microbe interactions, both at the cellular and at the molecular level, we therefore turned our attention to quantitative analysis of EIS recording and then to expression levels of certain genes. As with any potentiometric measurement, applied voltage causes ions to move through the tissue model. The structure of the tissue, the tight junctions in particular, impede the flux of ions, manifested as a retarded current and a higher impedance. The measurement is therefore a reflection of all the physical processes and structures that interact with ion flow. The true virtue of EIS is the fact that it is a linearization of this measurement, which allows for the contribution of each of these sources of impedance to be disambiguated. The most common approach to this goal is to model each impedance contribution as an equivalent circuit element; specifically, resistive intercellular junctions and capacitive cell membranes (bodies). Assembling an equivalent circuit in this way (including equivalent circuit elements which model the behaviour of the electrodes) allows for the parameter values of those elements to be fit by a complex‐valued regression. The most commonly used circuit model for an (epithelial) cell barrier is a resistance, *R*_b_, in parallel to a capacitance, *C*_b_, representing the impedance contributions of the paracellular and transcellular current (ionic flux) pathways respectively (as noted above). For this reason, we choose *R*_b_ as the figure of merit for barrier integrity, noting that it will be highly correlated to, but not the same as, conventional TEER (Figure S18, Supporting Information).

**Figure 4:**
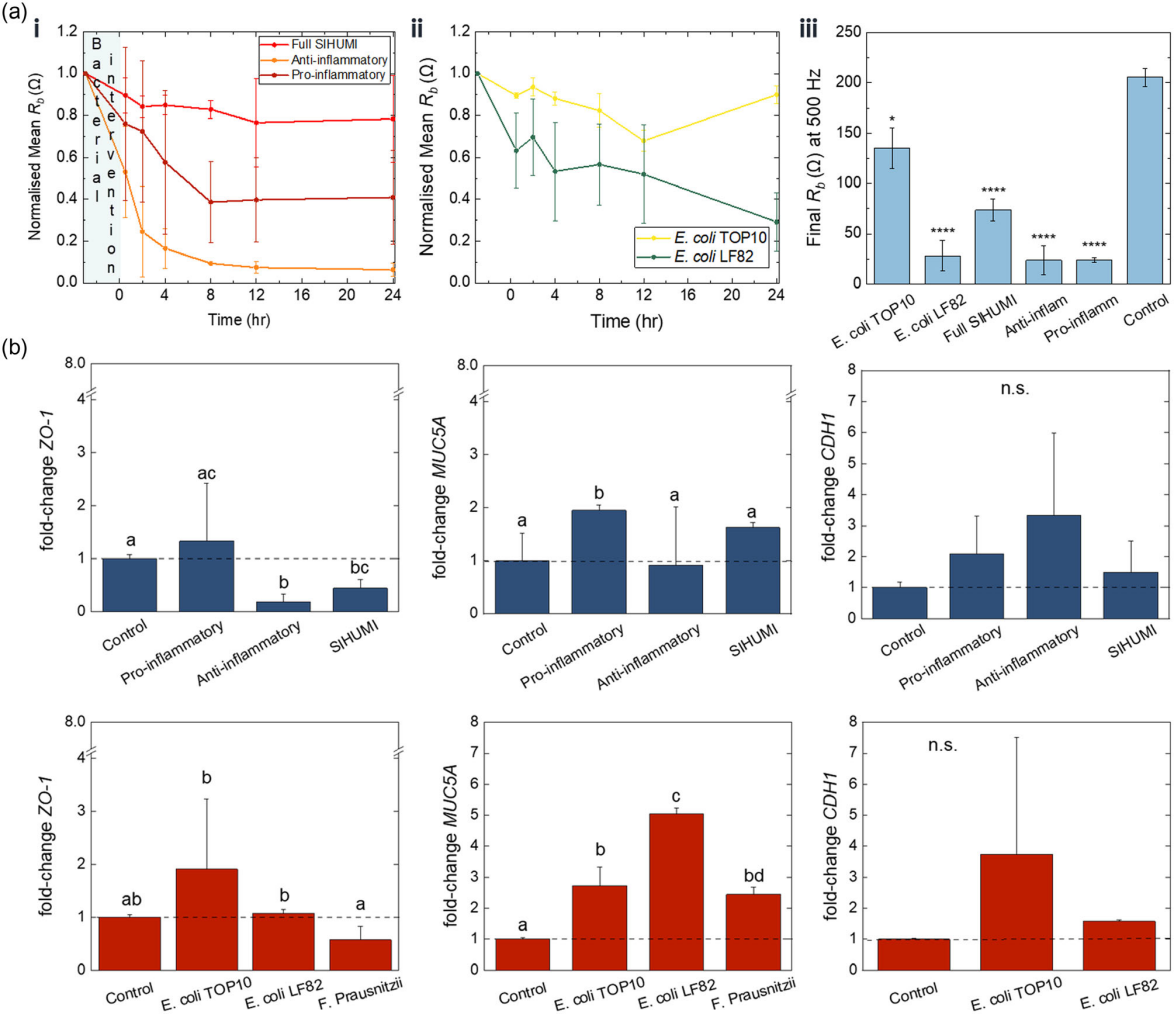
Quantitative analysis of gut barrier integrity upon postbiotic and live bacteria interventions. a) Evolution of *R*_b_ during the 24‐hour i) postbiotic and ii) live bacteria interventions. Note that *F. Prausnitzii* intervention was excluded from the analysis. Panel iii illustrates the mean fold‐changes of *R*_b_ ± SD of all interventions, relative to controls (no intervention). Each data point represents the mean *R*_b_ ± SD (*N* = 2, *n* ≥ 3), analysed by one‐way ANOVA, followed by Tukey's post‐hoc test (*…*p* < 0.05, **…*p* < 0.01, ***…*p* < 0.001, ****…*p* < 0.0001). b) Gene expression levels of gut barrier markers at the end of the 24‐hour interventions. Levels of mRNA expression of tight junction protein (ZO‐1), e‐cadherin adherence junction (CDH1) and mucin (MUC5A) are visualised as mean fold‐changes ± SD, relative to control samples (no intervention), from *N* = 2 biological replicates (*N* = 1 for *E. coli LF82* and *F. prausnitzii*) and *n* = 6–9 technical replicates, analysed by one‐way ANOVA on Ranks followed by Dunn's post hoc test. (*p* < 0.05). Different letters indicate statistical significance: values of each bar not sharing the same letters are significantly different at *p* < 0.05.

As seen in **Figure** **4**a, upon addition of each postbiotic and live bacteria cohort, *R*_b_ of the respective e‐Transmembrane gut tissue drops, as expected. In the case of pro‐inflammatory, anti‐inflammatory postbiotics, and *E. coli* LF82 *R*_b_ decreases rapidly from the first few hours post‐inoculation, reaching about 60%, 20% and 50% of the initial values, respectively. In contrast, SIHUMI and *E. coli* TOP10 induce a more limited decrease, with the *R*_b_ values reaching about 80% and 90% of the baseline levels, respectively. For the next few hours, *R*_b_ keeps decreasing in all interventions, although with different rates, mirroring the trends we observed in Bode plots above. Towards the end of the intervention experiment, *R*_b_ levels of tissues exposed to postbiotics appear to plateau, while the barrier integrity of tissues exposed to *E. coli* LF82 is further compromised. Importantly, devices exposed to *E. coli* TOP10 exhibit signs of reversing the initial drop of *R*_b_, reaching final values ≈90% of the values prior to intervention, as we have previously seen in a host‐microbe‐parasite interactions study using hydrogel‐based intestinal models.^[^ ^75^ ^]^ At the end of the 24‐hour monitoring, e‐Transmembrane intestinal barriers exposed to all six interventions exhibit statistically significant different *R*_*b*_ levels compared to control tissues (i.e., e‐Transmembrane intestinal tissues not exposed to interventions) (Figure 4a,iii). In parallel, we exposed IEC monolayers (3:1 Caco‐2/HT29‐MTX cells), grown and maintained in Transwell inserts, to the same 24‐hour postbiotic and live bacterial interventions and monitored their TEER using commercially available equipment. Although Transwell‐based monolayers exhibit similar response to the respective interventions in e‐Transmembrane intestinal tissues, changes in TEER were more limited in the case of, *E. coli* LF82, proinflammatory and full SIHUMI cohorts (Figure S19, Supporting Information). In contrast, in the case of *E. coli* TOP10, we found a more dramatic decrease of the 2D monolayer TEER during the first hours of exposure to bacteria, compared to the respective response of the 3D e‐Transmembrane tissue. In addition, while both 2D and 3D tissues exhibit recovery of the barrier integrity by reversing the initial drop of TEER and *R*_*b*_ respectively, e‐Transmembrane tissue reaches levels of integrity closer to its initial, before exposure to bacteria, values. A direct comparison between TEER and *R*_b_ is challenging due to both the biological features of the 2D and 3D tissues and the differences in the EIS setups. However, these findings suggest that our e‐Transmembrane platform and the electrical monitoring assays we developed are in line with current state‐of‐the art techniques.

To further investigate mechanisms of host‐microbe interactions at a molecular level, we screened for changes at the gene expression level of key gut barrier biomarkers: tight junction protein ZO‐1, adherens junction E‐Cadherin (CDH1) as well as the mucin protein MUC5A. To quantify gene expression, mRNA was isolated from all samples at the end of the 24‐hour interactions, including host tissue e‐Transmembranes that weren't exposed to a postbiotic or live bacteria intervention (i.e., control samples), and processed for qRT‐PCR using standard protocols. Fold‐changes of gene expression levels after each intervention, relative to control samples are summarised in Figure 4b. Overall, we can tell that postbiotics and live bacteria induce a differential effect on gene expression levels. In the case of postbiotics, there is a significant decrease in the level of genes related to ZO‐1 junction proteins of host tissues exposed to anti‐inflammatory and full SIHUMI cohorts. We also observed an increase in the fold‐change of CDH1 levels of tissues exposed to all postbiotics, but this was not statistically significant compared to control tissues. In the case of live bacteria interactions, ZO‐1 and CDH1 levels are higher in the presence of both *E. coli* strains, and in fact significantly higher for *E. coli* LF82 (for ZO‐1). ZO‐1 levels were also affected in the presence of *F. prausnitzii,* (we could not detect CDH1 in this case). Significant differences were also detected in the expression of mucin genes between postbiotic and live interventions. With the exception of anti‐inflammatory cohort, where a slight decrease was found, postbiotics induced a significant rise in MUC5A expression, with a ≈2‐fold change in the case of the pro‐inflammatory cohort and a ≈1.8‐fold change in the case of full SIHUMI cohort. Similarly, the pro‐inflammatory *E. coli* LF82 and *E. coli* TOP10 induced a statistically significant ≈5‐fold and ≈3‐fold increase respectively, whereas exposure of host tissue to *F. Prausnitzii* induced a ≈3‐fold increase, but this was not deemed statistically significant.

Taking a look now at up‐/downregulation of gene expression levels between postbiotics and their respective live counterparts (i.e., live bacteria species also present in the postbiotic cohorts), we first observe that both pro‐inflammatory postbiotics and *E. Coli* LF82 induce a statistically significant upregulation of MUC5A levels, which is more profound in the case of exposure to live pro‐inflammatory bacteria (≈5‐fold increase). Upregulation was also found in the case of ZO‐1 and CDH1 genes, but these changes were minimal. The anti‐inflammatory postbiotics and *F. prausnitzii* exhibit a similar trend in the case of ZO‐1 genes, decreasing their expression, but this downregulation was statistically significant only for the postbiotic intervention. The trend is opposite for MUC5A gene levels, where exposure to *F. prausnitzii* results in a profound upregulation, while the anti‐inflammatory postbiotics appear to not affect mucin expression.

## 4 Discussion and Conclusions

With the microbiome field rapidly expanding, several approaches have been employed to recreate the host‐microbiome niche and to dissect the interplay of its constituents in various conditions, as well to screen dietary supplements or pharmacological treatments of bacterial‐origin. Of particular interest are studies of dietary or therapeutic interventions that target the gut microbiota to support a healthy lifestyle and/or to ameliorate symptoms and treat intestinal diseases and disorders,^[^ ^81^ ^]^ such as probiotics and FMT. A great concern around such interventions, which holds back wide usage and translation to clinic, is safety, mainly because they contain live bacteria. Postbiotics have recently attracted a lot of attention for their potential to confer various health benefits to the host in a more controlled and safe manner. While the field is picking up pace quite fast, the mechanisms behind postbiotic effects on gut‐microbiome axis and host health remain poorly understood.

Pre‐clinical and clinical data, by nature, offer a ‘big picture’/broader perspective or insight at a large scale/whole‐body response of the aforementioned mechanisms under certain biological scenaria. In contrast, in vitro models, being reductionist, facilitate a closer observation and understanding of the mechanisms at play. Although both approaches are useful and insightful, the former offer complex and difficult to translate/interpret datasets, while the latter represent, at least until recently, far too simplistic models. However, organs‐on‐chips and microphysiological systems now offer the possibility to increase the complexity of in vitro models and to generate more relevant to human physiology data. Host‐microbiome studies, among other applications, have benefited from such advancements, with more and more gut‐/intestine‐on‐chip models and bioengineered tissues being employed to recreate the gut‐microbiota niche.^[^ ^40^, ^82^ ^]^ But there remain a few challenges to be addressed before this technology realises its full potential. Among them is the reliance of in vitro systems on end‐point assays, which limits their potential in accurately capturing certain phenomena, such as the fast kinetics of bacterial‐host interactions. In this study, we attempt to overcome some of these limitations by employing a 3D human intestinal tissue coupled with in‐line sensing units for rapidly assessing its barrier integrity upon interaction with live or inanimate bacterial cohorts, with known anti‐inflammatory/pro‐inflammatory properties, derived from a simple, yet well‐characterised, microbiota model – the SIHUMI.

We successfully used the e‐Transmembrane device to establish and in‐depth characterise a long‐term culture of a 3D human intestinal model, comprising intestinal and stroma cells, in the electroactive scaffolds of the 3D bioelectronic platform. Our two‐stage engineering strategy, allowed the development and maintenance of a stratified tissue in which individual cell components auto‐organised as they would in vivo: the intestinal epithelial barrier, consisting of enterocytes and goblet cells, is anchored on a lamina propria‐like layer, which is a network of fibroblasts and the ECM proteins they deposit. In contrast to commonly used passive/inactive substrates in Transwell models, OoCs, and bioengineered tissues,^[^ ^83^ ^]^ the intrinsic conducting properties of our scaffolds allowed us to use them, not only as substrates for the gut tissue, but also as electrodes that intimately couple with the biological system of interest for high‐resolution electrical recordings. In fact, EIS measurements throughout the experimental period (≈one month) provided continuous monitoring of tissue integrity, showcasing the benefits of using conducting polymers in in vitro tissue engineering.

Furthermore, our in‐line sensing assays allowed us to capture in real‐time, in a label‐free and non‐destructive manner, the rapid effects of inanimate and live bacteria on the e‐Transmembrane barrier tissue integrity, closely following the changes they induce in the tight junction‐regulated paracellular pathway during the 24‐hour microbial interventions. In fact, we established a framework for screening host‐microbe interactions in the e‐Transmembrane platform: 1) rapid qualitative evaluation of tissue integrity via recording impedance magnitude (Bode plots in Figure 2 and 3) and 2) quantification of breaches in the gut barrier tissue induced by microbiota activities by modelling the impedance spectrum with an equivalent circuit, fitting the circuit parameters and extracting those parameters pertaining to barrier function. Importantly, we show for the first time data on the operation of our devices in anaerobic conditions, when the respective tissue was exposed to *F. prausnitzii*.

Our impedance and *R*_b_ findings indicate clear differences between host‐postbiotic and host‐ live bacteria interactions, as well as between cohorts and between live species, which are mirrored in the confocal microscopy and molecular biology assays. However, direct correlations between electrical and optical readouts with gene expression levels to identify/validate the mechanism of host‐microbe cross‐talk are challenging, mainly due to the nature of each assay and the different spatiotemporal scales of the phenomena they describe: EIS measurements represent a measure of barrier integrity at the cellular/tissue level output, while mRNA expression findings reflect the host response at the molecular level, upon bacterial interventions. Identification of the exact mechanisms of cross‐talk was also limited by the number of biomarkers we screen for, as well as by the fact that the postbiotic and live bacteria preparation contents and the composition of supernatants, both before and at the end of the interventions, are not defined. For example, we expect that upon heat‐inactivation postbiotic preparations will contain internal antigenic molecules and lipopolysaccharides (LPS), which, in contrast to other endotoxins, would not be inactivated, given their resistance to heat treatments.^[^ ^84^, ^85^ ^]^ Moreover, we postulate that the LPS profile of SIHUMI cohorts would be significantly different, given that LPS is the major component of the wall of its Gram negative bacterial members (*E. coli and P. vulgatus).* To test this hypothesis and to overcome this limitation of the present study, we are now working on incorporating metabolomics and transcriptomics, the results of which could facilitate better understanding of the complex niche and interactions recreated in our e‐Transmembranes. In addition, although scarce, findings from in vivo studies would also be informative, validating the trends observed here.

Moving forward, a significant part of our efforts is now centred around improving the physiological relevance of our biological systems and the design, operation and outputs of e‐Transmembranes. Although the biological system we use here emulates key aspects of the complexity and functionality of its in vivo counterpart, it is derived from only three types of immortalised human cell lines. To enhance the physiological relevance of bioengineered tissues and, thus, to be able to generate more meaningful and translational data, we are working on establishing human intestinal organoid‐derived e‐Transmembrane gut models, as well as co‐cultures with other important cell/tissue types, such as immune cells and neurons. Another key parameter that is missing from the present study is shear stress, which has been shown to enhance the morphology and function of in vitro epithelial and endothelial tissues.^[^ ^86^ ^]^ Along with testing e‐Transmembranes and optimising their operation under microaerophilic and anaerobic conditions for further gut‐microbiota studies, we are also focused on incorporating fluidics in the platform. This would not only significantly enhance the biomimicry of our bioengineered tissues, but it could also improve the throughput of our studies.

Overall, here, we demonstrate a novel method for studying host‐microbiome interactions in an advanced bioengineered‐bioelectronic platform. Although this framework was developed in the context of screening highly‐relevant dietary/therapeutic microbiome‐derived candidates – postbiotics – it could be applied to various other applications. Our efforts are shifting towards modelling other tissues within our bioelectronic platforms, such as the lung and the blood‐brain‐barrier, as well as validating them as tools for drug screening and disease modelling studies.

## 5 Experimental Section

5.1

5.1.1

##### Device Fabrication

The scaffolds and device were prepared according to protocols previously reported,^[^ ^69^ ^]^ after slight modifications. Briefly, PEDOT: PSS aqueous dispersions (Clevios PH‐1000, Heraeus) were mixed with 0.5% w/v 4‐dodecylbenzenesulfonic acid (Sigma‐Aldrich) and 3% v/v 3‐glycidoxypropyltrimethoxysilane (Sigma‐Aldrich) and filtered (0.8 μm; Sartorius). The solution was ultrasonicated following each addition for 20 min with the temperature maintained between 5 and 10 °C. The resulting mixture was pipetted into a 48‐well plate (Thermo Scientific BioLite, cell‐culture treated) at 400 μl per well. The dispersion was kept at −20 °C for 24 h to promote ice‐templating, (i.e., a uniform and rapid formation of ice crystals in each well throughout the mixture). Post the pre‐cooling treatment, the aqueous dispersion in the well plates was freeze‐dried (VirTis Advantage Plus), with a dual freezing rate protocol. The freezing rate was set to 0.3 °C min^−1^ up to the point of crystallization (1 °C), after which a faster freezing rate of 0.9 °C min^−1^ was applied to a minimum temperature of −40 °C. The drying phase involved a temperature rise to 20 °C over 10 h at a vacuum pressure of 80 mbar. Prior to further processing, the scaffolds were annealed at 65 °C for 4 h to promote cross‐linking.

##### Cell Culture Maintenance

All cell lines were maintained at 37 °C and 5% CO_2_, under humidified conditions. Appropriate cell culture consumables were used for expanding, maintaining, and cryopreserving cells. Caco‐2 cells and HT29‐MTX cells were purchased from ECACC and were routinely maintained before all experiments. TIFs, labelled with red fluorescent protein (RFP–TIF LifeAct) were a gift from Ellen Van Obberghen‐Schilling, Institut de Biologie de Valrose). Caco‐2 cells (P54‐57) and HT29‐MTX‐E12 (P54‐58) cells were cultured in Advanced DMEM (Gibco), supplemented with 10% Fetal Bovine Serum (FBS, Sigma‐Aldrich), 1% m Glutamine (Glutamax‐1; Invitrogen), 1% penicillin‐streptomycin (10 000 U mL^−1^, Gibco), and 0.1% Gentamicin (Sigma‐Aldrich), seeded using the appropriate culture flasks at the recommended density of 20 000 cells cm^−2^. TIF LifeAct (P9‐12) were cultured in Advanced DMEM (Gibco, Life technologies) supplemented with 20% FBS (Sigma Aldrich), 1% Glutamine (Gibco, Life technologies), 2% HEPES (Gibco, Life technologies), 1% penicillin–streptomycin (10 000 U mL^−1^, Gibco, Life technologies), and 0.1% Gentamicin (Sigma Aldrich). All cells were harvested with 0.25–0.5% trypsin‐prior to seeding or passaging.

##### Establishment of Gut Barrier Tissue in Transwells and in e‐Transmembranes: Development of Intestinal Cell Monolayers in Transwell Inserts

To generate intestinal monolayers in Transwell inserts we followed the method described here.^[^ ^75^ ^]^ Briefly, a ratio of 3:1 Caco‐2 and HT29‐MTX coculture, (i.e., IEC coculture) was seeded on the top of Transwell membranes (Greiner Bio‐One ThinCert hanging cell culture inserts, 0.33 cm^2^ growth area, PET membrane, Cat no 662 630), at a density of 20 000 cells cm^−2^, after obtaining and mixing the appropriate number of each cell type in a single cell suspension of the desired ration. Cells were maintained for 21 days in complete growth medium, as described above, with media replacements every other day.

##### Establishment of Gut Barrier Tissue in Transwells and in e‐Transmembranes: Device Preparation for Seeding

This experimental step is based on previous work of our group, as described here.^[^ ^68^, ^69^ ^]^ Briefly, on the day of the experiment and after the e‐Transmembrane inserts are assembled and placed in dedicated wells of a 24‐well plate, the complete platform undergoes thorough sterilisation with 70% ethanol. To remove and deactivate ethanol, sterilisation is followed by careful and thorough rinsing of the inserts, wells and lid sterile DI water and PBS. Each e‐Transmembrane device is then carefully immersed in complete growth media of TIFs, (as this is the first cell type seeded in the scaffolds), to allow for protein adhesion on the scaffold surface during a two‐hour incubation. Note that media is also added in the apical compartment, so that the top electrodes (i.e., reference/CE) are fully immersed in each well, which is necessary for obtaining electrical readouts. After the incubation, we obtain the first electrical measurements (see below), and then each insert is carefully rinsed with fresh, pre‐warmed complete growth medium of TIF cells and then transferred carefully in a dedicated well of a non‐treated 12‐well plate for seeding.

##### Establishment of Gut Barrier Tissue in Transwells and in e‐Transmembranes: Tissue Engineering Strategy/Cell Seeding

We have previously reported on a two‐stage tissue engineering strategy to generate a stratified gut barrier tissue, comprising a connective tissue‐/lamina propria‐like layer and an intestinal epithelial layer.^[^ ^68^, ^69^ ^]^ At the first stage, 250 000 TIF RFP LifeAct cells, suspended in 50ul of complete growth medium, are seeded in each e‐Transmembrane device, by carefully dropping the cell suspension on the apical surface of their PEDOT:PSS scaffolds (places in the non‐treated 12‐well plate as described above). Cells seeding is followed by incubation for two hours, after which each e‐Transmembrane is carefully transferred back to its dedicated well of a sterile (treated/cell culture grade) 24‐well plate, pre‐filled with warm, fresh TIF RFP LifeAct growth media. Media is also added in the apical compartment, as before. The 3D culture generated at this stage are routinely maintained for 4 days to allow for development of a 3D network of fibroblasts and ECM, naturally deposited by this cell type. Electrical readouts are also obtained (see below).

The second stage of the tissue establishment strategy takes place on experimental day 5 and after obtaining an electrical readout (baseline; see below). First, medium from the apical compartment of each e‐Transmembrane insert is removed and the devices are carefully transferred to a sterile non‐treated 12‐well plate. A 50μl cell suspension of 250 000 Caco‐2 and HT29‐MTX cells (in a 3:1 ratio, respectively) is then seeded in each device and cells are incubated for two hours, as before. After this point, the devices are transferred back to dedicated wells of a sterile (treated/cell culture grade) 24‐well plate, pre‐filled with warm, fresh tri‐culture medium (i.e., cell growth medium the composition of which is adjusted to accommodate growth/maintenance of all cell types, see here^[^ ^68^ ^]^). The 3D culture system generated here is maintained for three more weeks (total culture period: 26–27 days). Media changes take place three times a week, after obtaining electrical readouts (see below).

##### Preparation of Inanimate and Live Bacterial Cultures and Interface with Host Tissue: Preparation of Postbiotic Cohort Inocula

The strains *Escherichia coli* LF82*, Ruminococcus gnavus* ATCC 29 149*, Enterococcus faecalis* OG1RF*, Bifidobacterium longum* ATCC 15 707*, Faecalibacterium prausnitzii* A2‐165 and *Phocaeicola vulgatus* DSM 1447 *(*formerly *Bacteroides vulgatus)* were cultivated in cultured in Wilkins‐Chalgren Anaerobe Broth (Oxoid) at 37 °C in Hungate tubes to maintain anaerobicity. To validate that anaerobic conditions were maintained the media was supplemented with (1 mg L^−1^) resazurin as an indicator of oxygen presence. After overnight growth, bacterial numbers were assessed by plating on Wilkins‐Chalgren Anaerobe Agar (see Table 1) and then each culture (i.e., 6 individual strains and the full cohort) were thermally inactivated (i.e., autoclaved at 121 °C for 15 mins). Prior to the interventions, respective strains for the pro‐inflammatory and anti‐inflammatory cohorts are mixed in the ratios they typically fall into in the full SIHUMI cohort.^[^ ^74^ ^]^

##### Preparation of Inanimate and Live Bacterial Cultures and Interface with Host Tissue: Preparation of Live Bacteria Inocula

*Escherichia coli* TOP10 GFP cells (a gift from Dr Graham Christie's lab) were grown in LB broth, supplemented with 100 μg mL^−1^ carbenicillin (Sigma‐Aldrich). *Escherichia coli LF82* cells (APC Microbiome Ireland) were grown in Mueller–Hinton broth, supplemented with 100 μg mL^−1^ carbenicillin (Sigma‐Aldrich). After an overnight incubation at 37 °C with shaking at 125 rpm, each bacterial strain culture was diluted 1:100 in fresh broth and grown until they reached mid‐exponential phase. Bacterial growth was monitored spectrophometrically at an optical density of 600 nm (OD_600_). *Faecalibacterium Prausnitzii* A2‐165 (DSMZ, Germany) cells were recovered from frozen for 24 h and then a 1:100 dilution of the stock culture was grown in Yeast Casitone Fatty Acids Broth with Carbohydrates Broth (YCFAC; Anaerobe Systems, USA) within an anaerobic cabinet (Whitley Workstation DG250, UK), until the culture reached exponential phase (≈10 h; Figure S13c, Supporting Information).

On day 22 of intestinal cell culture (day 27 of total experimental period in the case of e‐Transmembranes), each bacterial strain inoculum of 0.25 OD_600_ was prepared from liquid cultures at mid‐exponential phase. *E. coli LF82* and *E. coli TOP10* inocula were pelleted and then washed twice with sterile PBS and resuspended in modified growth medium, consisting of Advanced DMEM, 1% Glutamine, 2% FBS, and 0.5% antibiotics (1:1 gentamycin:penicillin‐streptomycin; see here for more details^[^ ^75^ ^]^). Preparation of *F. Prausnitzii* inoculum was carried out via the same steps, however, the modified growth medium in which this inoculum was resuspended was deoxygenated in an anaerobic chamber for 48 hrs prior to use.

Each inoculum was then added in the apical surface of dedicated Transwell inserts and e‐Transmembrane devices and maintained at 37 °C in a humidified incubator for 24 h. Devices interfaced with *F. Prausnitzii* were handled in a glove box and maintained in a chamber under anaerobic conditions (0.1% O_2_, 37 °C, 5% CO_2_; (PhO2x Box, Baker Ruskin). During the co‐culture assay, along with electrical monitoring (see below), Transwell inserts and devices were frequently checked for contamination visible by naked eye (e.g., color and transparency of media). Please note that inspection with an inverted microscope is not possible due to the presence of reference/CE electrodes in the lid above each e‐Transmembrane well.

##### Imaging: Confocal Microscopy

All samples were fixed in 4% paraformaldehyde (PFA, ThermoFischer Scientific) for 10–12 min, at room temperature, followed by thorough washing with PBS. Scaffolds were then carefully removed from the inserts and stored at 4 °C until ready to use for staining. Prior to immunostaining, cells were permeabilized in 0.1% v/v Triton X‐100 (Fisher) for 10 min and then blocked for nonspecific binding with 1% wt/v BSA (Fisher) and 0.1% v/v Tween‐20 (Fisher) in PBS for 1 hr at room temperature. The primary and secondary antibodies as well as all labeling kits used are: Phalloidin‐iFluor 594 Reagent (abcam), Rabbit polyclonal anti‐ZO‐1 (ThermoFischer Scientific), Mouse monoclonal anti‐MUC2 (abcam), Rabbit Anti‐Laminin 1 + 2 (abcam), Mouse Anti‐Fibronectin (abcam), Mouse Anti‐Collagen I (abcam), Goat‐anti‐rabbit Alexa Fluor 488 (abcam), Goat‐anti‐mouse Alexa Fluor 647 (abcam), Bisbenzamide H (Hoechst 33 342) (abcam). Slices were then placed on microscopy plates and were kept hydrated with PBS during imaging. Images were obtained using an epifluorescence/confocal microscope (Axio Observer Z1 LSM 800, Zeiss), using 10×/0.45, (Plan‐Apochromat, Zeiss) and 20×/0.8, (Plan‐Apochromat, Zeiss) objectives. At least two independent samples were visualized for each condition. The 2D micrographs shown are representative of three different frames/locations of each, while z‐stacked images were acquired from one region of each independent sample.

##### Imaging: Histological Analysis

Sections of the e‐Transmembrane scaffolds (≈30 μm) were stained for vimentin (VM; Dako MAB Clone V9), for epithelial cytokeratins (CK; Dako MAB Clone MNF116), and for mucin with Alcian Blue (1% in 3% acetic acid; TCS chemicals) and Periodic Acid Shiff (PAS; periodic acid Surgipath, schiffs; Atom scientific) techniques, following routine protocols. Please note that each section was stained for one of the aforementioned markers.

##### Imaging: Scanning Electron Microscopy

Samples were fixed with 2.5% glutaraldehyde (Fisher BioReagents) in PBS overnight followed by dehydration in a graded ethanol series and then in hexamethyldisilazane solution (HDMS, Sigma Aldrich) until complete dehydration as follows: Samples were immersed in 50% ethanol and then 70% ethanol, each for 1.5 h at −20 °C, followed by further dehydration at 95% ethanol and then 100% ethanol, each for 1 h at 4 °C. HMDS and ethanol solutions at a ratio of 1:2 followed by a 2:1 ratio was added for 20 min each at room temperature. Pure HMDS was subsequently added and allowed to evaporate at room temperature overnight. To obtain sagittal cross‐sections of the e‐Transmembrane scaffolds, samples were sliced vertically with a scalpel and mounted onto carbon adhesion tapes on the surface of aluminium stubs. A 25 nm carbon coating was applied by sputtering (Combined Coater System, Agar Scientific) before imaging on a FEI Nova NanoSEM 450 (HD: 5 kV).

##### Electrical Monitoring: Transwell‐Based Intestinal Models

In the case of Transwell‐based intestinal models, the integrity of intestinal barriers was evaluated by dynamically monitoring the TEER of the barriers, provided as a single output from the CellZscope device (Nanoanalytics GmbH), which measures the frequency‐dependent impedance of barrier‐forming cell cultures grown on permeable supports. Based on our previous studies,^[^ ^75^ ^]^ on day 22, before postbiotic or live bacteria inoculations, Transwells were transferred in dedicated wells of the CellZScope multi‐well units and were allowed to adjust for ≈4 h inside a humidified incubator at 37 °C and 5% CO_2_. After this, a baseline measurement (i.e., integrity of the barrier before inoculation assays) was acquired automatically. Following this, postbiotic and live *E. coli* TOP10 or LF82 were prepared as described above and added in the apical compartment od dedicated Transwells. Impedance measurements were then taken automatically via the CellZscope software at pre‐determined time points; first measurements 30 and 60 mins post‐inoculation and then every 2 h. TEER values for each sample and for each time point were directly extracted from the CellZScope software.

##### Electrical Monitoring: e‐Transmembranes

Electrochemical impedance spectroscopy was performed using a potentiostat (Metrohm Autolab) equipped with a frequency response analysis module. As described before^[^ ^69^ ^]^ and according to the geometry of the device (see Figure 1), a two‐electrode electrochemical cell was created, with the scaffolds on the gold (Au) base electrode, acting as the working electrode (WE) and a Pt mesh (20 mm × 20 mm) as the counter electrode (CE), fitted in the lid of the well plate and fully immersed in the apical compartment medium (+/‐ inanimate or live bacteria inocula). Please note that using the potentiostat in a two‐electrode electrode configuration, equates to using CE as the reference electrode (RE). The distance between CE and WE remained the same for all the measurements. The applied AC voltage amplitude was 0.01 10 mV_RMS_, and measurements were carried out at a bias voltage of 0.1 V_{vs RE}_ (equating to an applied DC voltage of −0.1 V_{CE‐WE}_) DC potential versus open circuit potential. The frequency was swept from 100 kHz to 100mHz, recording 10 points per decade. Measurements were obtained prior to changing media in the culture during the ≈4 week growth of the tissue. Measurements were taken in the same way during the 24‐hour incubation of host tissue with bacterial inocula. Devices interfaced with *F. Prausnitzii* were handled and measurements were taken in the glove box under hypoxic conditions. Simulation and fitting of the curves were carried out using a bespoke MATLAB application. Circuit parameters were fit by the complex non‐linear, least squares method using MATLAB's multistart, global optimization routine, with the series resistance, *R*_electrolyte_, fixed to the real part of the highest frequency impedance measurement. The circuit model used was (see Figure S18, Supporting Information): (*R*_CE_//*Q*_CE_)—*R*_electrolyte_—(*R*_barrier_//*C*_barrier_). Where ‘//’ denotes a parallel combination and ‘—’ denotes a series connection, and 'CE’ denotes the counter electrode (and implicitly accounts for the working electrode/scaffold impedance). *R*_name_, denotes a resistance associated with a named circuit element. Similarly, *C*_name_, denotes a capacitance with impedance $Z_{C}={\left(j\omega C\right)}^{-1}$and, *Q*_name_, denotes a constant phase element, with impedance $Z_{Q}={\left(Q{\left(j\omega \right)}^{\alpha }\right)}^{-1}$.

##### Quantitative qRT‐PCR

Regulation of the mRNA expression of the selected gut barrier genes induced by postbiotics and live bacteria was detected in the 2D Transwell and the 3D bioelectronic gut model after 24 h incubation time. RNA was isolated using TRIzol Reagent (Thermofisher, UK). RNA isolation, cDNA synthesis and PCR were performed using Monarch Total RNA Miniprep Kit, LunaScript RT SuperMix Kit and Luna Universal qPCR Master Mix (New England BioLabs, UK). Real‐Time PCR was carried out with a QuantStudio 6 Pro Real‐Time PCR System. Primers were validated by sequencing the obtained PCR product (Eurofins Genomics Germany GmbH, Germany) (See **Table** **2**).

**Table 2:** Primer sequences used for target and housekeeping genes.^[^ ^87^, ^88^, ^89^ ^]^

Species	Gene	Gene ID	Forward 5′‐3′	Reverse 5′‐3′
Target genes				
human	ZO‐1	7082	TGCCATTACACGGTCCTCTG	GTTGATGATGCTGGGTTTGTTT
human	MUC5A	4586	TATGTGCTGACCAAGCCCTG	TTGATCACCACCACCGTCTG
human	CHD1	999	CCCGCCTTATGATTCTCTGCTCGTG	CTGTAATCCCAATACTCTGGGAGGC
Housekeeping genes				
human	GAPDH	2597	GCACCGTCAAGGCTGAGAAC	ATGGTGGTGAAGACGCCAGT
human	HPRT1	3251	TGACACTGGCAAAACAATGCA	GGTCCTTTTCACCAGCAAGCT

##### Statistical Analysis

Data were analyzed using OriginPro, Version 2021 (OriginLab Corporation, Northampton, MA) for Windows. Each data point represents the mean ± standard deviation of two independent experiments (*N;* biological replicates) with at least three e‐Transmembrane devices (*n;* technical replicates). For the establishment of the gut tissue in the e‐Transmembranes *N *= 2 and *n* = 8. For the intervention studies, *N* = 2, *n* = 3–6 devices per timepoint (*N* = 1 for live *F. Praus*) For the Transwell monolayer model interventions *N* = 1 and *n* = 4 inserts per condition and timepoint. Homogeneity of variance was evaluated via a Levene's test (*p* > 0.01). Statistical comparison between multiple groups was analyzed using a one‐way analysis of variance (ANOVA), followed by Tukey's post‐hoc test. See figure legends for *p*‐values to deem statistical significance in each test. mRNA expression was calculated using the LinRegPCR, Version 2021.1 (Academic Medical Centre, Amsterdam, the Netherlands) and using GraphPad Prism 9.4.1 software (GraphPad Software, Boston, MA). Statistical significance of two biological and 6–9 technical replicates for postbiotic and live *E. coli* TOP10 interventions and of one biological replicate and 6–9 technical replicates for *E. coli* and *F. prausnitzii* live bacterial interventions was tested by one‐way analysis of variance (ANOVA) followed by Dunn's post‐hoc test Different letters indicate statistical significance: values of each bar not sharing the same letters are significantly different, a = control e‐Transmembrane tissues (not exposed to any intervention) (*p* < 0.05).

## Conflict of Interest

The authors declare no conflict of interest.

## Author Contributions

C.‐M.M. and R.M.O. conceived of the study. C.‐M.M., D.v.N., and L.A.D. developed the methodology. C‐M.M. designed and performed the experiments, analysed and visualised the data. C.P. and D.v.N. fabricated the devices and D.v.N. worked on modelling of the EIS data. V.S. performed the qRT‐PCR experiments and data analysis and cultured *F. praus* bacteria. A.M.W. assisted with cell culture maintenance and qRT‐PCR assays. L.A.D. cultured bacteria and prepared the postbiotics. K.H. supported with the histopathological staining and analysis of images. R.M. acquired the SEM images. R.A. assisted with the fabrication of the scaffolds. C.H. contributed to the design of the study and data interpretation. C.‐M.M. and R.M.O. wrote the manuscript. All authors reviewed and edited the manuscript. R.M.O. supervised the project and secured funding.

## Code Availability Statement

Simulation and fitting of the impedance curves were carried out using a bespoke MATLAB application, available at Apollo, the University of Cambridge repository at the following link: https://doi.org/10.17863/CAM.102234.

## Supporting information

Supplementary Material

## Data Availability

The data that support the findings of this study are openly available in [Apollo, the University of Cambridge repository] at [https://doi.org/10.17863/CAM.102234], reference number [102234].
