# Supplementary material for: Modelling Human Gut‐Microbiome Interactions in a 3D Bioelectronic Platform
Source: Small Sci. 2024 Apr 22;4(6):2300349. doi: 10.1002/smsc.202300349 (PMC11935216; doi:10.1002/smsc.202300349)
Supplement: Supplementary file 1 — Supplementary Material [file SMSC-4-2300349-s001.pdf]

Supporting Information

Modelling human gut-microbiome interactions in a 3D bioelectronic platform

Chrysanthi-Maria Moysidou, Douglas van Niekerk, Verena Stoeger, Charalampos Pitsalidis, Lorraine A. Draper, Aimee M. Withers, Katherine Hughes, Reece McCoy, Rachana Acharya, Colin Hill, Róisín M. Owens

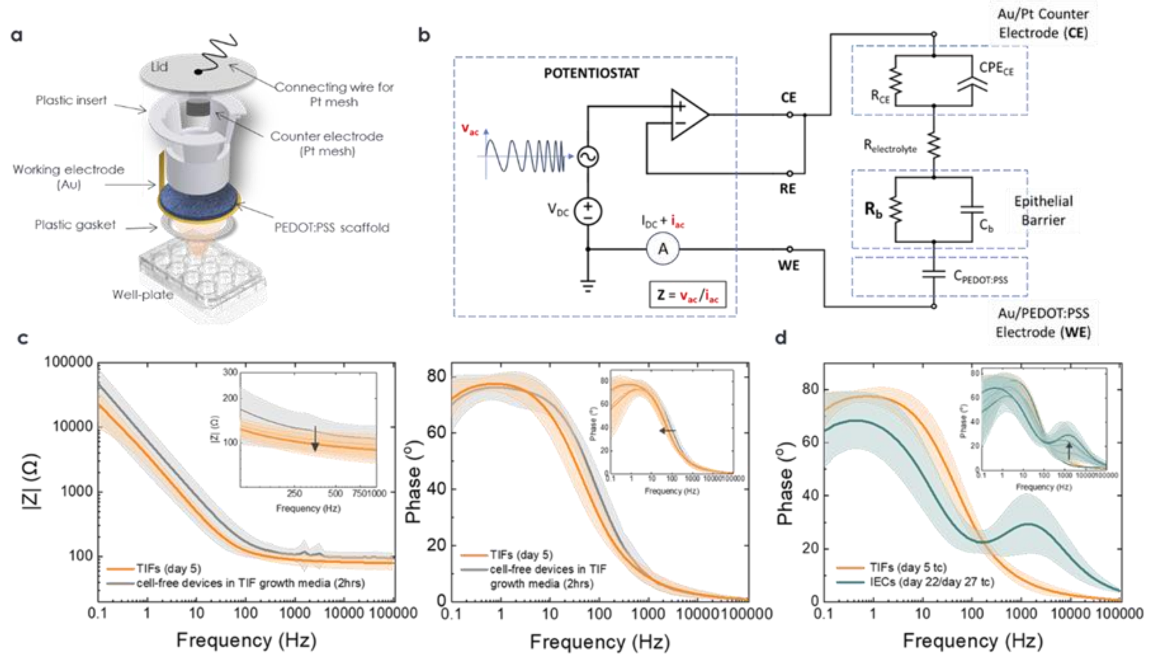

Supplementary Figure 1: **a** Schematic illustrating the components of the e-Transmembrane device. Additional EIS output features of the e-Transmembranes during tissue establishment. **b** Equivalent Circuit Model and instrumentation setup used for the extraction of R_b. The measurement is conducted by way of a potentiostat, connected in the two-electrode configuration (reference electrode terminal shorted to the counter electrode terminal) and operating in constant voltage, measured current (potentiostatic) mode. A small-signal voltage frequency sweep, superimposed atop a DC bias voltage is applied to the device (CE vs WE) and the resulting current is measured. The ratio of the small signal (alternating) voltage to the small signal component of the measured current yields the impedance. **c** Bode plot of the mean impedance magnitude and corresponding Phase of the e-Transmembranes prior to cell seeding and after the 2hr – incubation with TIF complete growth medium, in grey, and the respective mean impedance of 5-day TIF-cultured scaffolds (prior to IEC seeding), in orange. The respective colour bands indicate the SD (*N=2, n=8*); **d** Phase plots corresponding to the impedance BODE plot of the devices in Fig.1B. The orange line corresponds to the mean value of the Phase of the fibroblast-cultured devices, prior to IEC seeding (day 5 tc), and the green line corresponds to the mean value of the Phase upon establishment of the stratified intestinal tissue, at the end of the experimental period (day 27 of total culture (tc)). The respective colour bands indicate the SD (*N=2, n=8*).

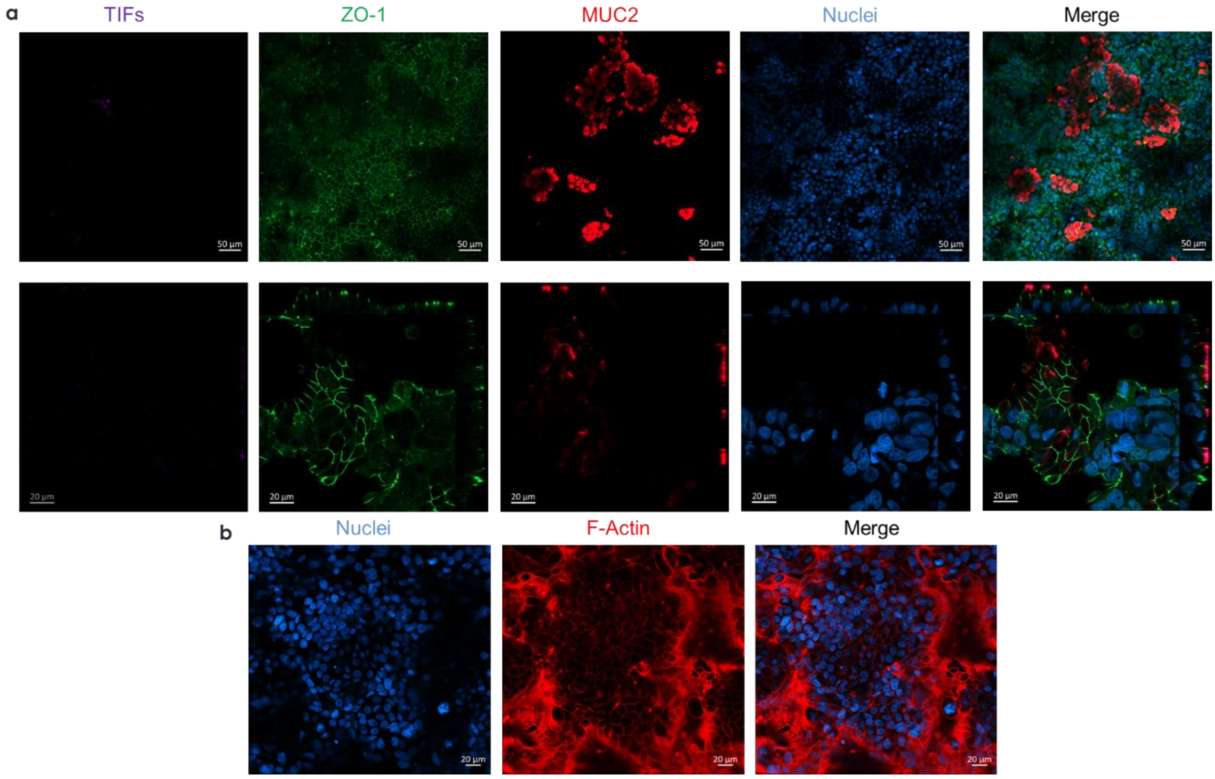

Supplementary Figure 2: Confocal images of the barrier tissue apical domain in e-Transmembranes. Panels correspond to individual channels and merged channels of the confocal images shown in **a** Fig. 1C and **b** Fig. 1D, revealing establishment of tight junction network (ZO-1 in green), with the typical chicken-wire pattern, mucin (MUC2 in red; panels in A) secretion in the apical surface of the layer, and filamentous actin cytoskeletal network (F-acting in red; panels in b), all counterstained for nuclei.

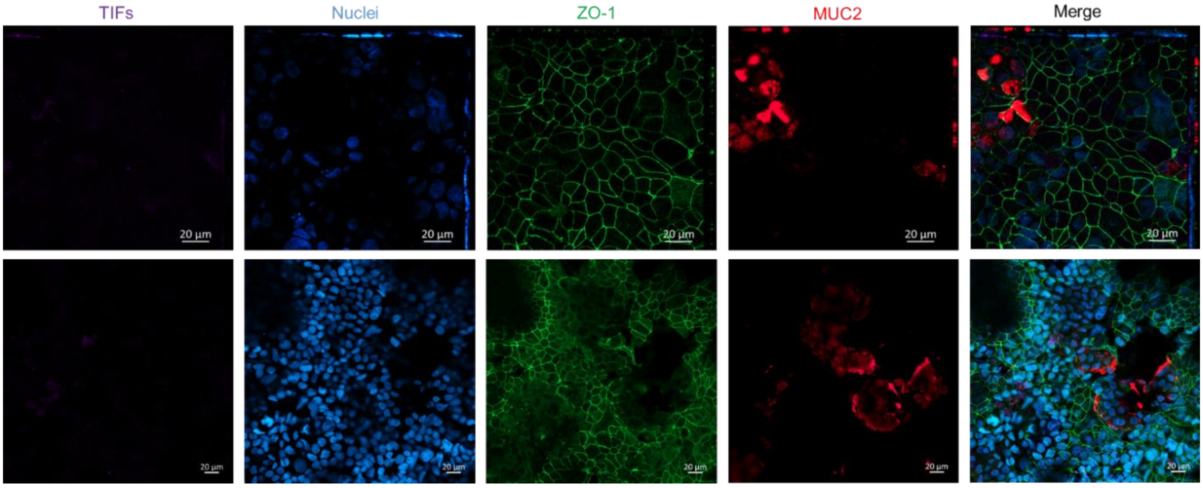

Supplementary Figure 3: Additional confocal images of the barrier tissue apical domain in the e-Transmembranes. Panels correspond to individual channels and merged channels of confocal images of samples stained for ZO-1 tight junction (green) and MUC2 (red) proteins, counterstained for nuclei (blue).

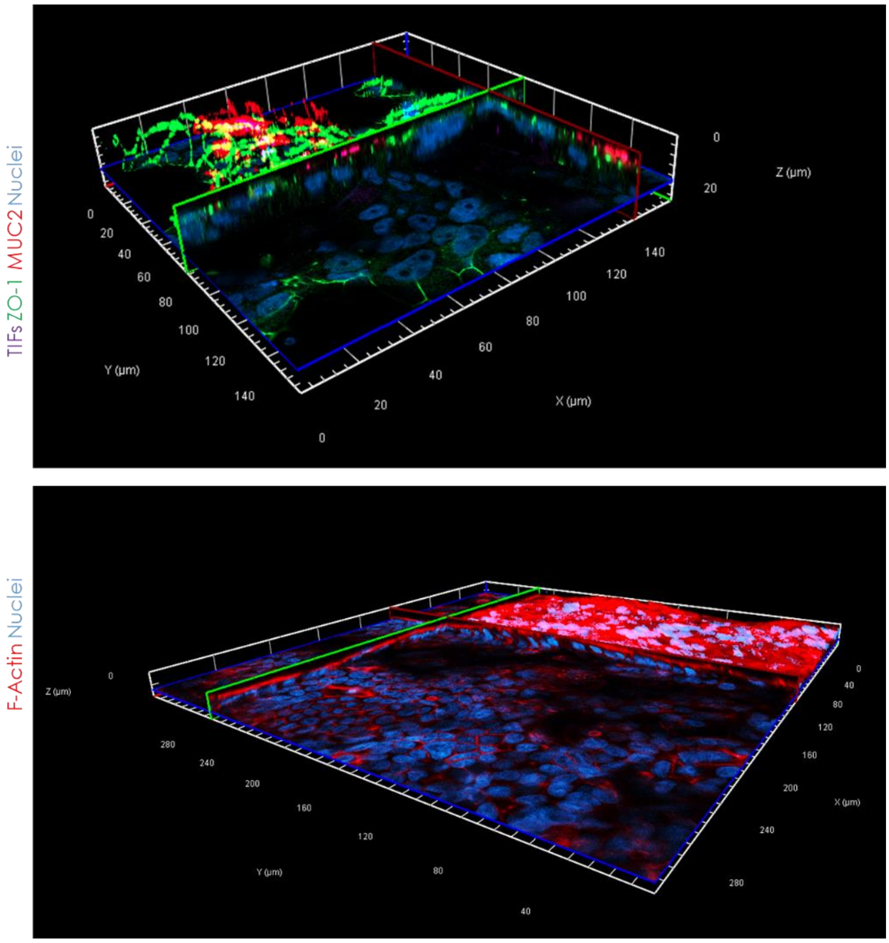

Supplementary Figure 4: 3D renderings of z-stacked confocal images of e-Transmembrane tissue samples, illustrating a section of the continuous epithelial layer formed by the IECs of the tri-culture system of the 3D model, stained for ZO-1 tight junctions (green), MUC2 protein (red) and nuclei (blue) in the top panel and filamentous actin (red) and nuclei (blue) in the bottom panel.

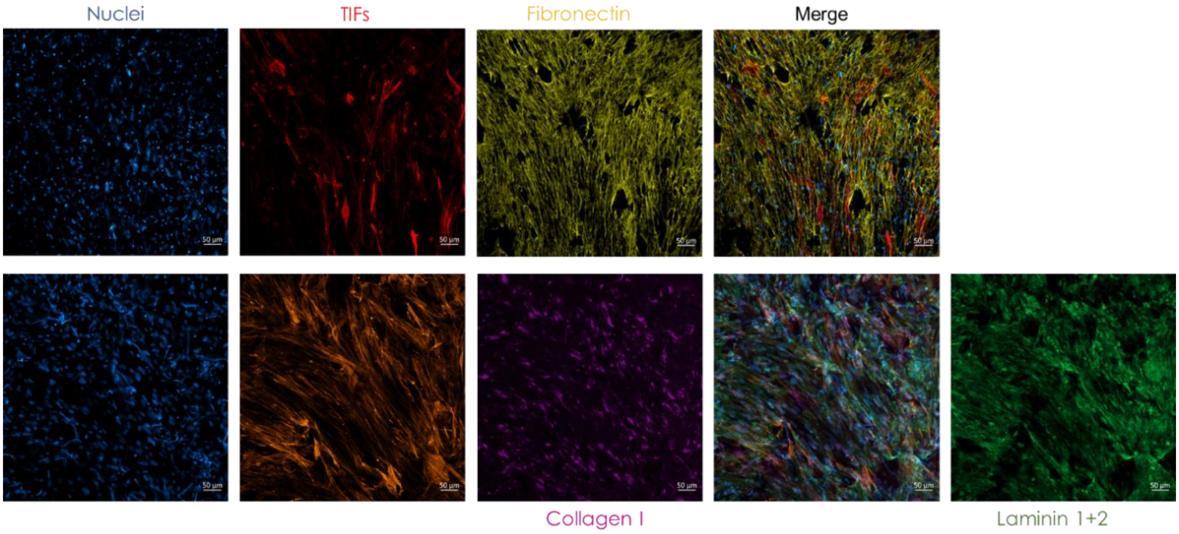

Supplementary Figure 5: Confocal images of the barrier tissue basal domain in e-Transmembranes. Panels correspond to individual channels and merged channels of the confocal images shown in Fig. 1F, revealing the formation of a lamina propria-like layer by fibroblasts and the ECM proteins (fibronectin in yellow, collagen in purple, laminin in green) they deposit in the bulk of the electroactive substrates. Samples were counterstained for nuclei (blue).

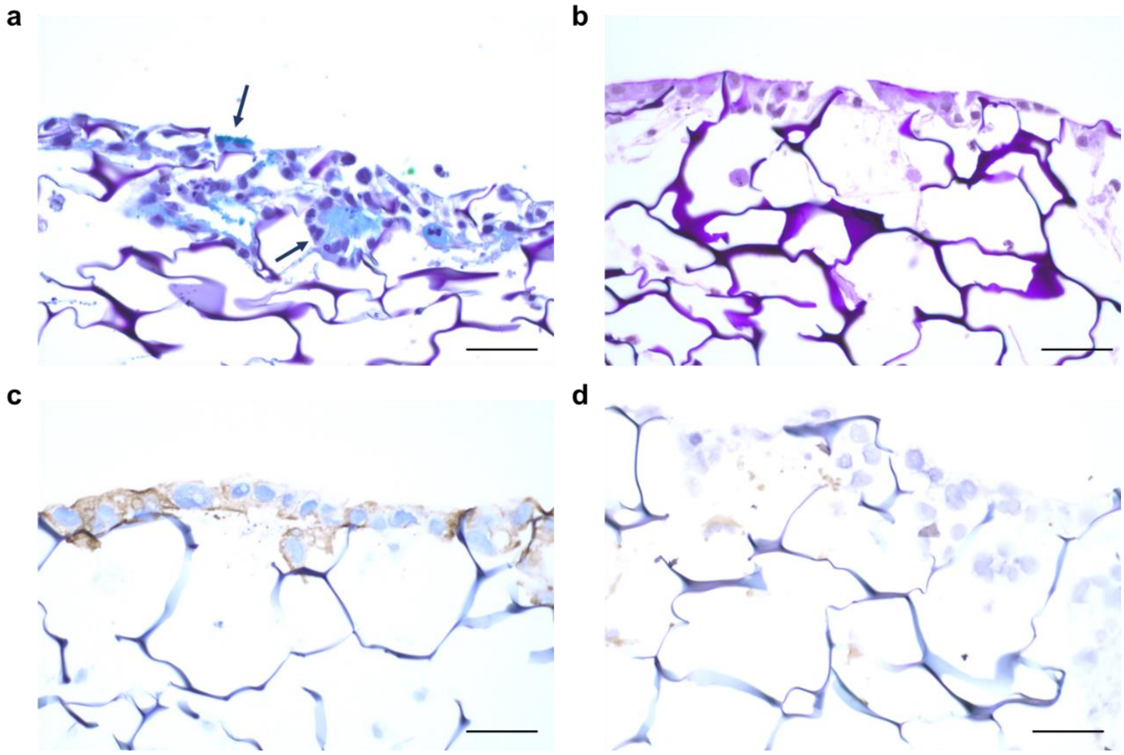

Supplementary Figure 6: Morphological characterisation of the cell self-organisation towards formation of the stratified intestinal tissue in the electroactive scaffolds of the e-Transmembranes. Histopathological staining for **a** Alcian blue (indicating mucin production (blue arrows) and differentiation of cells), **b** PAS, **c** Pan-cytokeratin and **d** vimentin. Note that each slice/section was stained for the specific biomarker, which explains why the pores of the scaffolds appear empty (absence of cells) and in some cases or the intestinal monolayer is not visible. Scale bar 40µm.

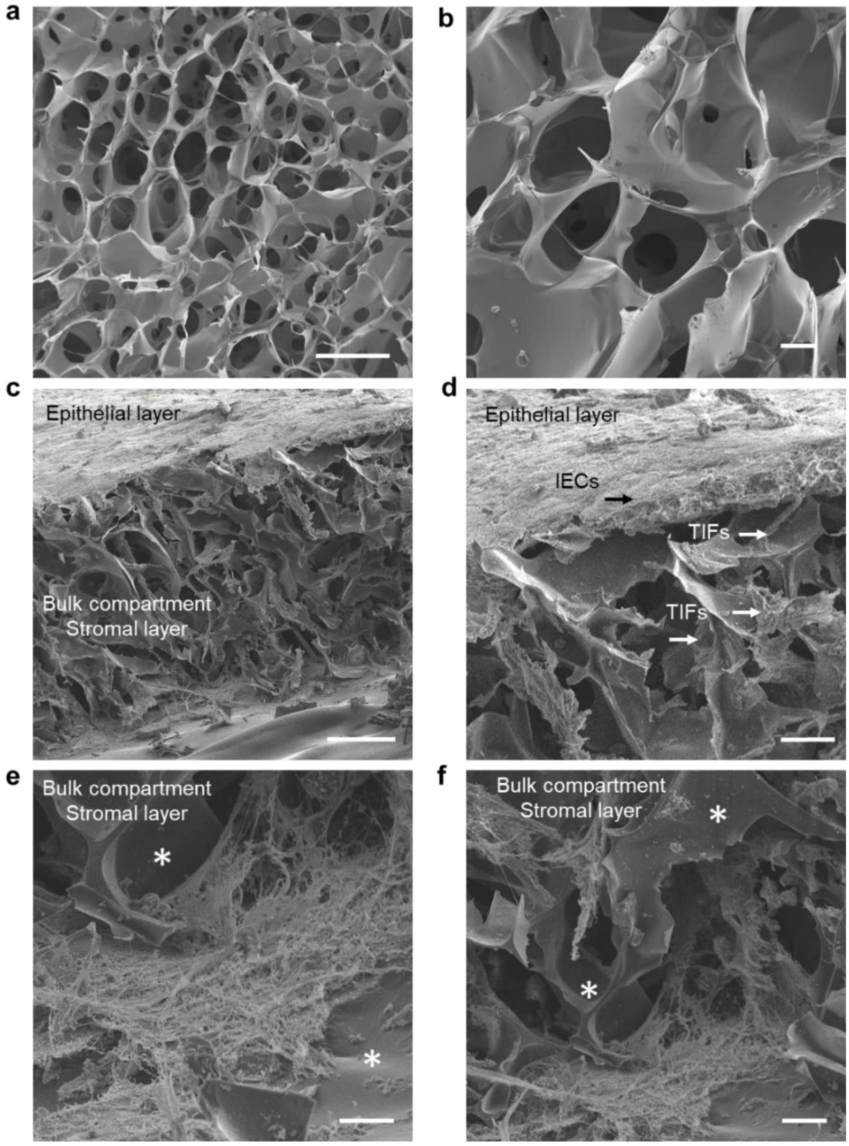

Supplementary Figure 7: Morphological characterisation of the establishment of the intestinal and lamina propria layers of the stratified intestinal tissue in the electroactive scaffolds of the e-Transmembranes. Scanning Electron Microscopy (SEM) micrographs of **a,b** cell/tissue-free e-Transmembrane scaffolds (scale bars: 100µm and 20µm) and **c-f** of e-Transmemebrane scaffolds hosting the stratified intestinal tissue at the end of the 4-week experiments. Image **c** illustrates a sagittal cross-section of the e-Transmembrane scaffolds, revealing a monolayer of intestinal epithelium on the apical surface, supported by a thick layer of fibroblast-derived stromal tissue layer

in the bulk compartment of the scaffolds (scale bar: 200µm). Image **d** is a magnified version of image **c**, offering a closer look at the interface between the two tissue layers (scale bar: 50µm). White arrows indicate representative locations of TIF/ECM deposition in the scaffolds. Images **e-f** offer a closer look at the fibroblast infiltration of the bulk compartment, revealing the network established by fibroblast and the ECM they secrete and deposit within the scaffold porous network, giving rise to the stromal tissue layer of the stratified intestinal tissue of e-Transmembranes (scale bars:10µm and 20µm). The wall of the scaffolds is annotated with white asterisks.

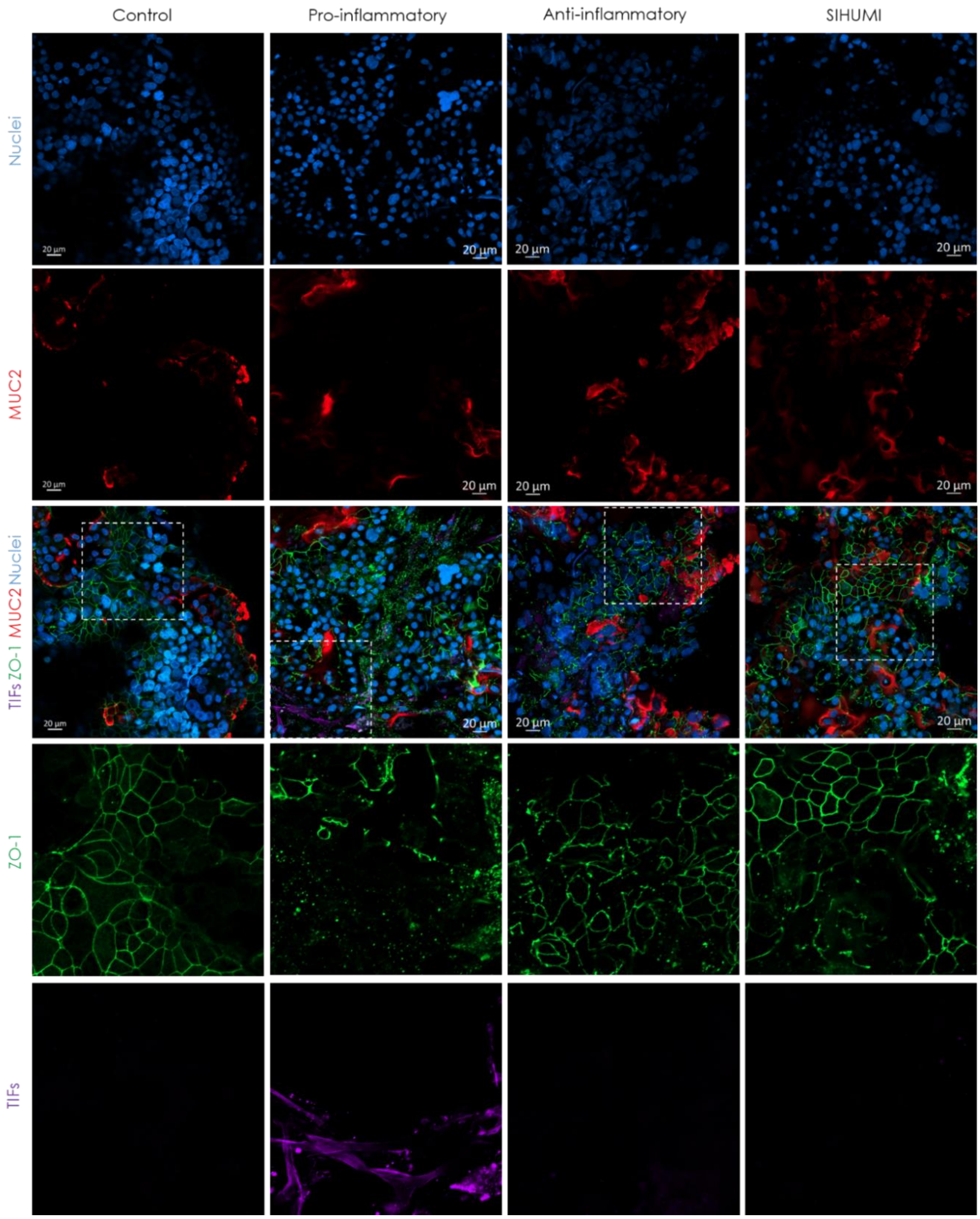

Supplementary Figure 8: Additional confocal images (for Fig. 2C) of the barrier tissue apical domain in the e-Transmembranes exposed to postbiotic interventions. Panels correspond to individual channels and merged channels of confocal images of control, pro-inflammatory, anti-inflammatory and SIHUMI samples stained for ZO-1 tight junction (green) and MUC2 (red) proteins, counterstained for nuclei (blue), also showing the RFP-labelled TIFs (here switching the colour to purple for better visualisation).

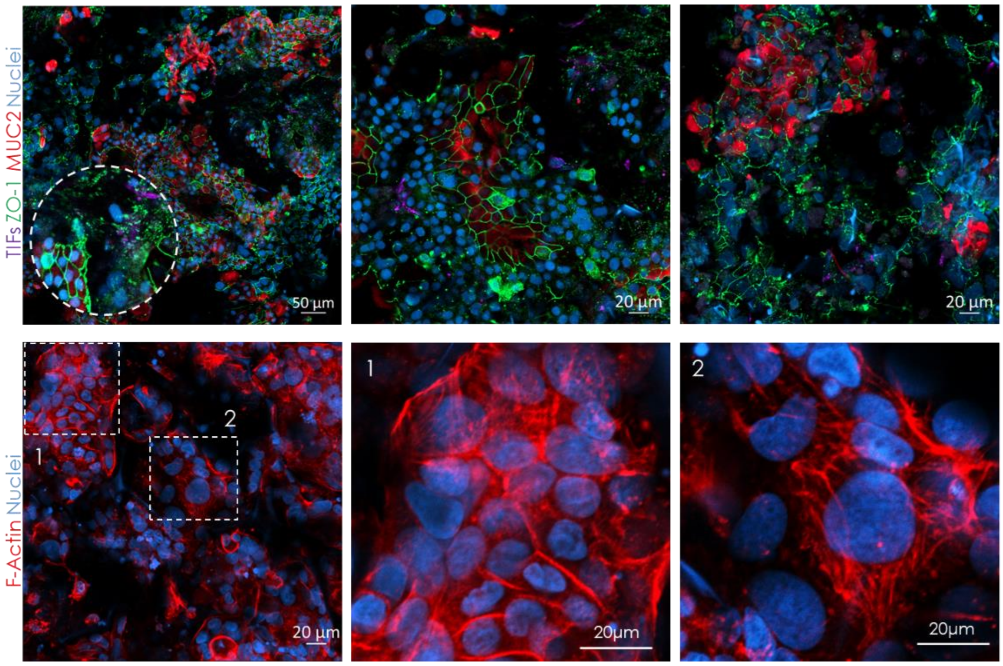

Supplementary Figure 9: Additional confocal images of the barrier tissue apical domain in the e-Transmembranes exposed to pro-inflammatory postbiotics. Top row illustrates immunofluorescence staining for ZO-1 tight junction (green) network and MUC2 (red) proteins, counterstained for nuclei (blue), and the RFP-labelled TIFs (here switching the colour to purple for better visualisation). Round dashed annotated area is a close-up, revealing a disturbed epithelial layer. Bottom row panels illustrate immunofluorescence staining of samples for F-actin (red) and nuclei (blue). Images annotated as 1 and 2 are close-ups of the corresponding annotated areas in the left panel.

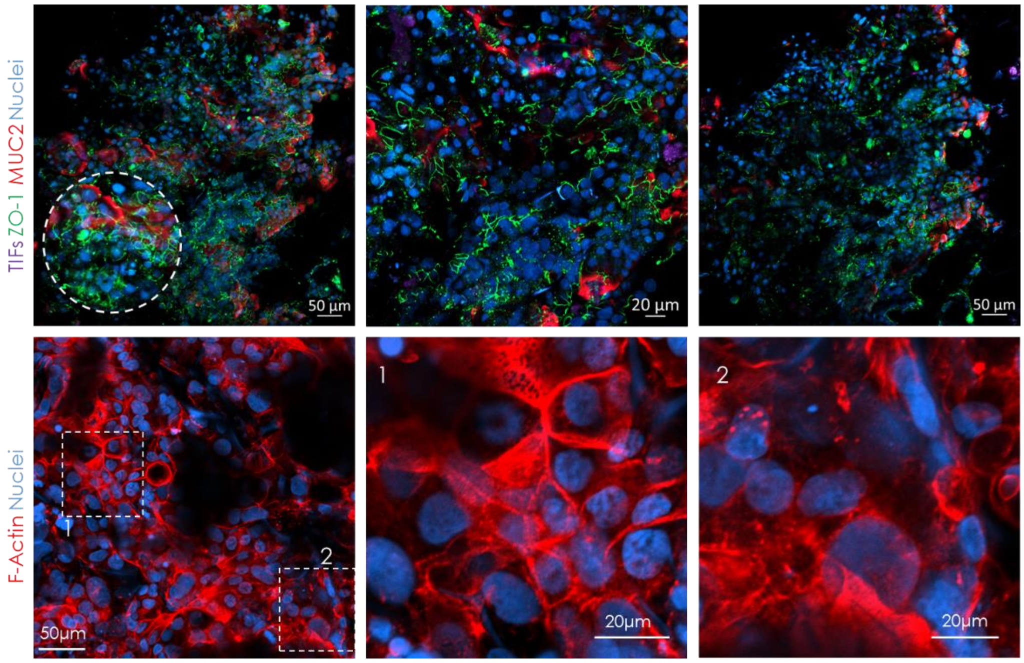

Supplementary Figure 10: Additional confocal images of the barrier tissue apical domain in the e-Transmembranes exposed to anti-inflammatory postbiotics. Top row illustrates immunofluorescence staining for ZO-1 tight junction (green) network and MUC2 (red) proteins, counterstained for nuclei (blue), and the RFP-labelled TIFs (here switching the colour to purple for better visualisation). Round dashed annotated area is a close-up, revealing an impared epithelial layer. Bottom row panels illustrate immunofluorescence staining of samples for F-actin (red) and nuclei (blue). Images annotated as 1 and 2 are close-ups of the corresponding annotated areas in the left panel.

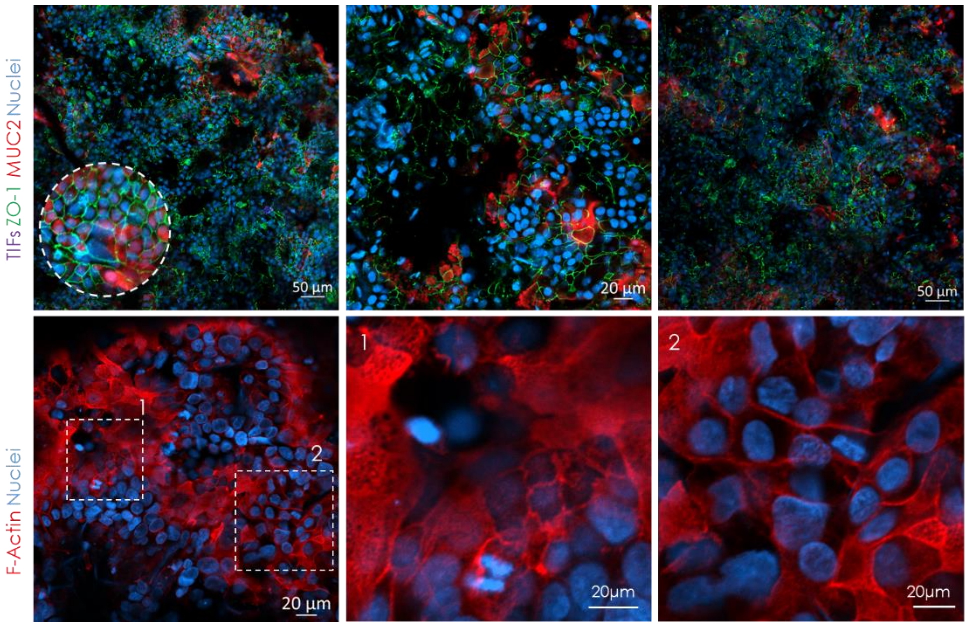

Supplementary Figure 11: Additional confocal images of the barrier tissue apical domain in the e-Transmembranes exposed to SIHUMI postbiotic cohort. Top row illustrates immunofluorescence staining for ZO-1 tight junction (green) network and MUC2 (red) proteins, counterstained for nuclei (blue), and the RFP-labelled TIFs (here switching the colour to purple for better visualisation). Round dashed annotated area is a close-up, revealing a slightly disturbed epithelial layer. Bottom row panels illustrate immunofluorescence staining of samples for F-actin (red) and nuclei (blue). Images annotated as 1 and 2 are close-ups of the corresponding annotated areas in the left panel.

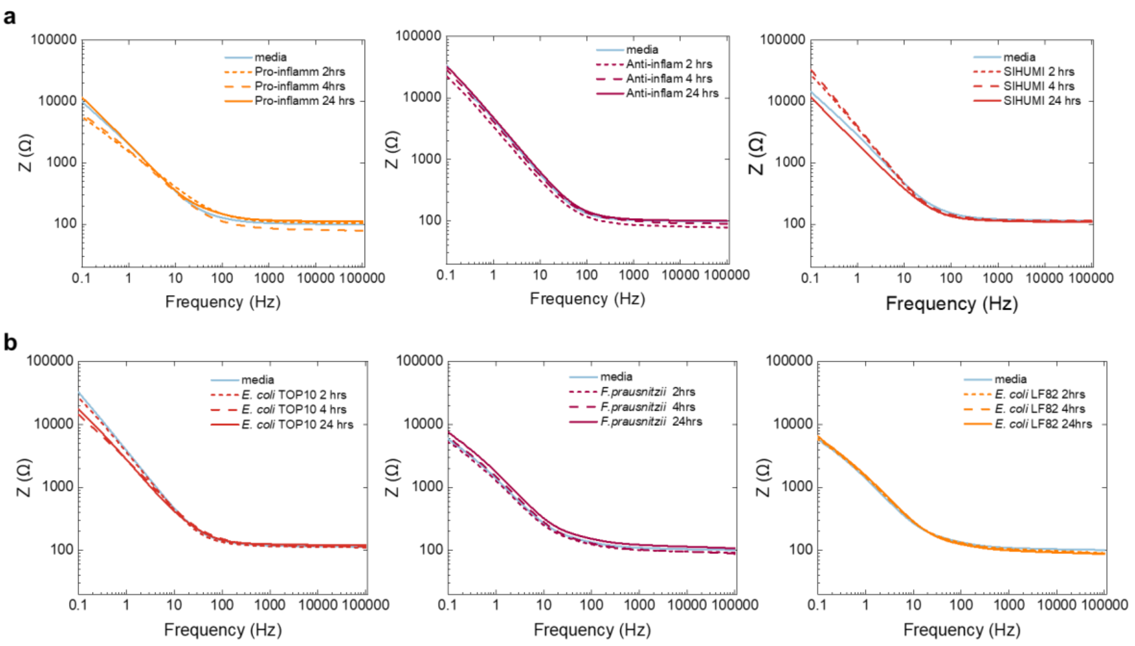

Supplementary Figure 12: Bode plots of host tissue-free/blank e-Transmembrane devices exposed to **a** postbiotic and **b** live interventions for 24-hours.

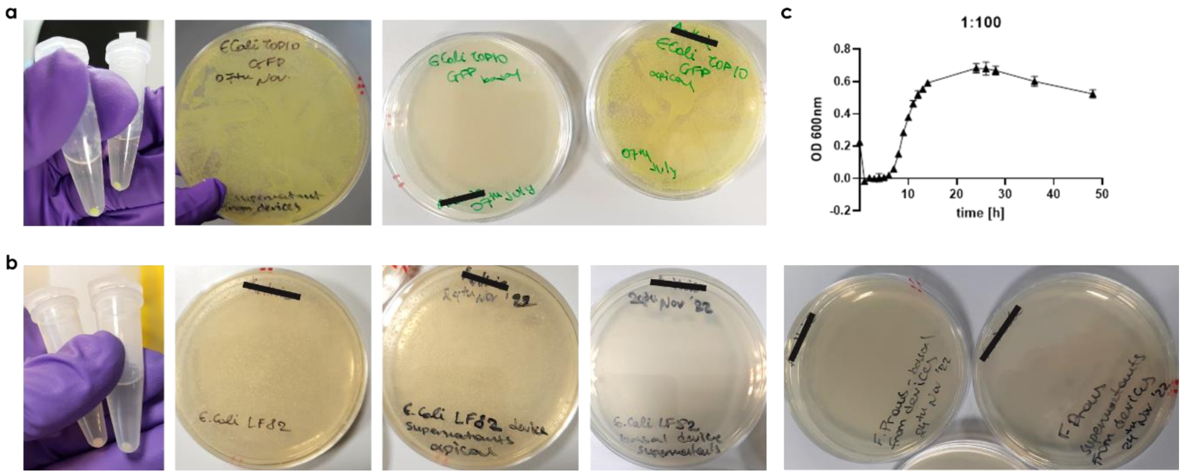

Supplementary Figure 13: **a** *E. coli TOP10* and **b** *E. coli LF82* viability after the 24-hr interaction with the host tissue model. **c** *F. Prausnitzii* growth curve (top) and viability after the 24-hr interaction with the host tissue model (bottom).

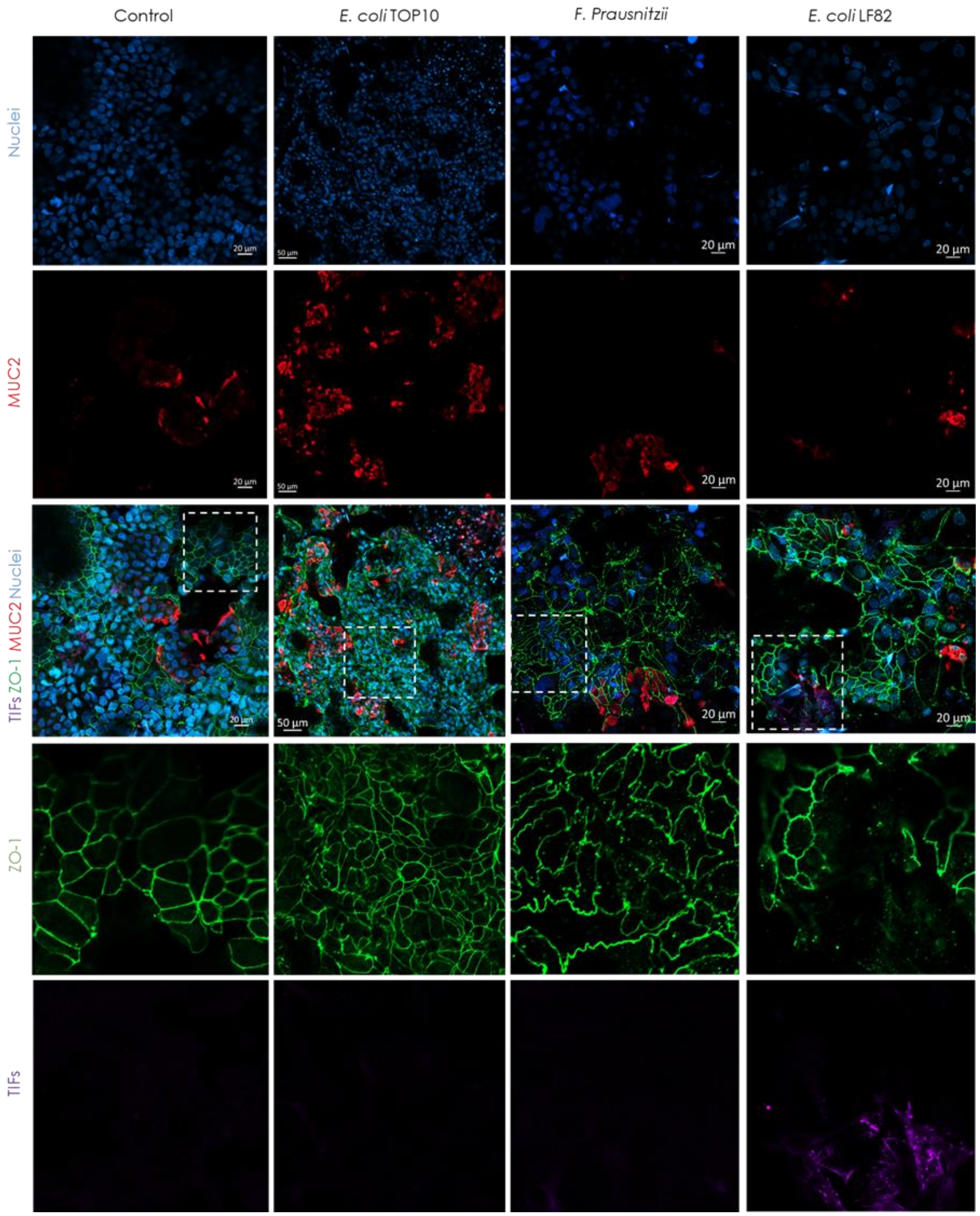

Supplementary Figure 14: Additional confocal images (for Fig. 3C) of the barrier tissue apical domain in the e-Transmembranes exposed to live bacteria interventions. Panels correspond to individual channels and merged channels of confocal images of control, *E. coli* TOP10, *F. prausnitzii* and *E. coli* LF82 samples stained for ZO-1 tight junction (green) and MUC2 (red) proteins, counterstained for nuclei (blue), also showing the RFP-labelled TIFs (here switching the colour to purple for better visualisation).

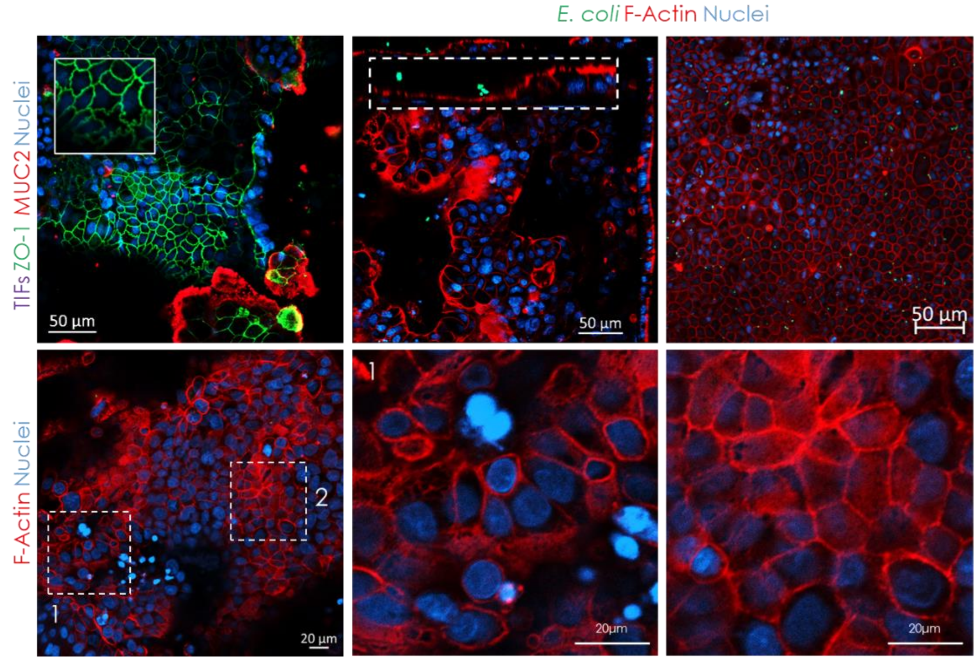

Supplementary Figure 15: Additional confocal images of the barrier tissue apical domain in the e-Transmembranes exposed to *E. coli* TOP10. Top left panel illustrates immunofluorescence staining for ZO-1 tight junction (green) network and MUC2 (red) proteins, counterstained for nuclei (blue). Annotated area is a close-up that shows a negligible disturbance of the TJ network upon interaction with bacteria. Top middle and right panels reveal interaction of the GFP-labelled *E. coli* with the F-actin-labelled epithelial tissue (in red). Annotated area in the middle panel is a close-up of the x/z plane of the corresponding ortho-view image, revealing adherence of the bacteria on the brush border of the barrier tissue. and the RFP-labelled TIFs (here switching the colour to purple for better visualisation). Round dashed annotated area is a close-up, revealing a slightly disturbed epithelial layer. Bottom row panels illustrate immunofluorescence staining of samples for F-actin (red) and nuclei (blue). Images annotated as 1 and 2 are close-ups of the corresponding annotated areas in the left panel.

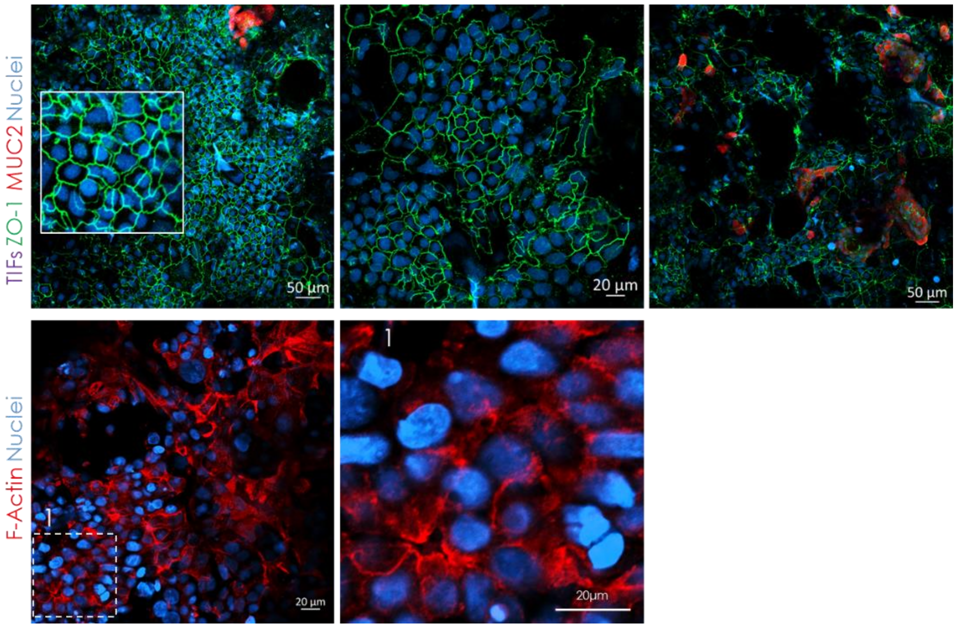

Supplementary Figure 16: Additional confocal images of the barrier tissue apical domain in the e-Transmembranes exposed to *F. Prausnitzii*. Top row panels illustrate immunofluorescence staining for ZO-1 tight junction (green) network and MUC2 (red) proteins, counterstained for nuclei (blue) and the RFP-labelled TIFs (here switching the colour to purple for better visualisation). Annotated are in right panel is a close-up, revealing the effect of the bacterium on TJ network morphology. Bottom row panels illustrate immunofluorescence staining of samples for F-actin (red) and nuclei (blue). Image annotated as 1 is a close-up of the corresponding annotated area in the left panel.

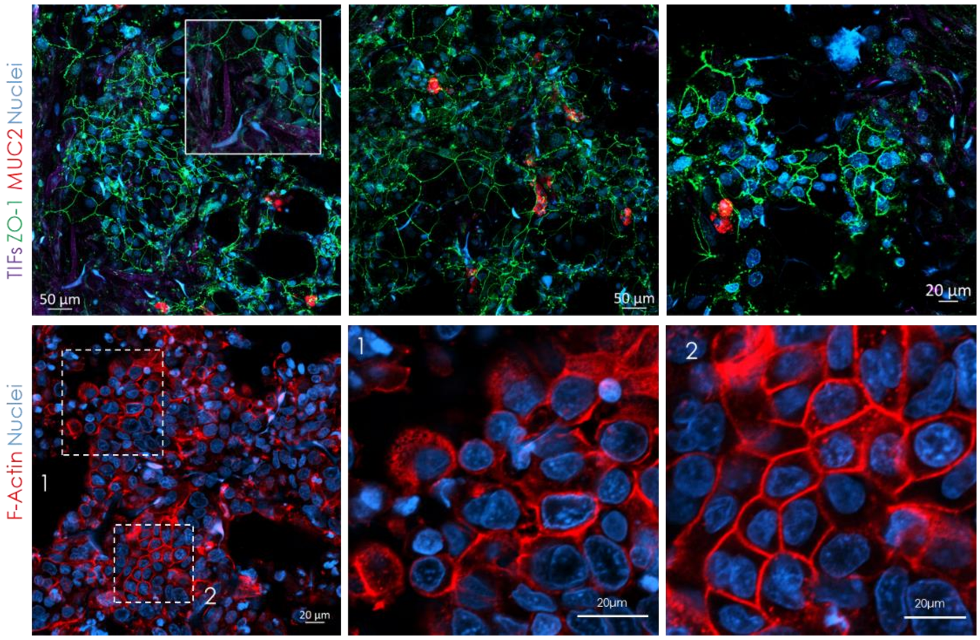

Supplementary Figure 17: Additional confocal images of the barrier tissue apical domain in the e-Transmembranes exposed to *E. coli* LF82. Top row panels illustrate immunofluorescence staining for ZO-1 tight junction (green) network and MUC2 (red) proteins, counterstained for nuclei (blue) and the RFP-labelled TIFs (here switching the colour to purple for better visualisation). Annotated are in right panel is a close-up, revealing the TIF-derived tissue layer underneath the disrupted epithelial layer. Bottom row panels illustrate immunofluorescence staining of samples for F-actin (red) and nuclei (blue). Images annotated as 1 and 2 are close-ups of the corresponding annotated areas in the left panel.

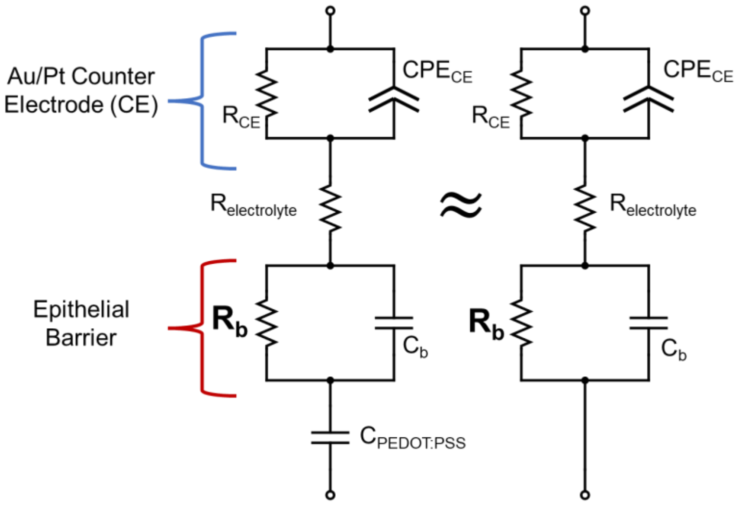

Supplementary Figure 18: The equivalent circuit considers the system as a linear combination of the counter (gold/platinum mesh) electrode, the electrolyte, the epithelial barrier and the PEDOT:PSS scaffold (working electrode). R_{CE} is a leakage resistance associated with the counter electrode, CPE_{CE} is a constant phase element modelling the counter electrode capacitance, $R_{electrolyte}$ models the conductivity of the cell growth medium, $C_{PEDOT:PSS}$ models the capacitance of the conducting polymer scaffold, C_b models the capacitance of the transcellular pathway and R_b models the resistance of the paracellular pathway. The equivalent circuit is simplified by approximating the PEDOT:PSS capacitance as a short circuit, as its contribution is negligible at sufficiently high frequencies. The circuit fitting in the estimation of R_b is therefore given on the right-hand side of the panel.

The conducting polymer, PEDOT:PSS is known to behave capacitively, where the capacitance scales with the volume of the polymer. As the volume of PEDOT:PSS in the scaffold is very large, the corresponding capacitance, $C_{PEDOT:PSS}$ is very large. Furthermore, the impedance of a capacitive element is given by $Z_C = (j\omega C)^{-1}$ and is inversely proportional to the capacitance. The counter electrode comprises of a gold pin in contact with a platinum mesh, which also behaves primarily capacitively, but scales with the surface area of the metal. The scaffold impedance is thus much smaller than the capacitance of the platinum mesh – given that they are in series, the larger element dominates. For sufficiently large frequencies, the impedance contribution of either capacitive element becomes negligible; the design of the device is such that the time constants associated with the electrodes and the time constant associated with the barrier are well separated (please see also the updated Supplementary Figure 1b). Therefore, at the mid and high frequencies, at which the barrier contribution is measurable, the electrode impedances are relatively negligible and can be excluded. While the capacitive electrodes necessarily contribute a 90° phase lag to the current, the net phase is the ratio of the real to complex components of the impedance and thus the large

barrier-impedance-to-electrode-impedance ratio at the frequencies of interest motivates for a negligible phase contribution by the electrodes in addition to the negligible impedance magnitude contribution. At low frequencies, the electrode impedances are appreciable and superimposed on the barrier resistance; the smaller capacitance of the counter electrode results in a relatively large contribution to the impedance magnitude and the net impedance approximates the counter electrode impedance alone. Furthermore, the series combination of the capacitive CE and capacitive WE confound the independent contributions of the two electrodes and the measurement and the impedance is equivalent to the measurement of a single, net capacitance, in series with the biological barrier. The superposition of the two capacitive elements, which contribute meaningfully to the impedance within the same frequency band, makes disambiguating the two electrodes challenging and including both electrodes in the model, without additional *a priori* information is ambiguous. In order to improve the robustness of the fit, we combine the capacitive contributions of the two electrodes in the equivalent circuit model, reducing the number of degrees of freedom in the regression.

The electrolyte (cell growth medium) is well known to behave resistively, however, it should be noted that $R_{electrolyte}$ models the irreducible series resistance in the system at large, which includes the electrolyte as well as the resistances of any wiring and connections used in the measurement setup. The impedance spectrum tends to $R_{electrolyte}$ at high frequencies. A standard model for the tissue barrier is used, where the paracellular pathway, namely the tight junctions between adjacent cells, is primarily resistive. The cells themselves, however, behave as capacitors, allowing charge accumulation at the apical and basal aspects of their membranes, resembling parallel plate capacitors. A less commonly used circuit configuration is employed for the counter electrode; the platinum mesh is used for the electrode. As an inert material, coupling between the electrode and electrolyte is restricted to electric double layers (EDLs), for modest applied potentials. EDLs are considered non-ideal capacitors, where the non-ideality is often accommodated for by substituting the capacitance with a constant phase element (CPE). Furthermore, the mesh construction causes the electrode to act as a porous material, which pushes the capacitive behaviour further from ideality. Ultimately the decision to use a CPE was justified empirically, as the counter electrode impedance is prominent in the low frequency regime, where it can be seen that the phase angle tends to a value <90°, characteristic of a CPE. A parallel resistance is added to the counter electrode model through similar observations of the impedance data, namely, the phase angle can be seen to peak and then decrease with decreasing frequency at the ultra-low frequency limit. This is indicative of a resistive leak element, in parallel to the electrode capacitance. It is likely that the gold pin, used to contact the platinum, is in contact with the electrolyte also. Gold is more likely to engage in redox reactions with the electrolyte, yielding a resistive pathway which bypasses the platinum electrode-

electrolyte interface. Indeed, the fitted values for R_{CE} are of the order of $10^5\ \Omega$ – given the small surface area of the gold and low operating voltage, this hypothesis seems the most likely.

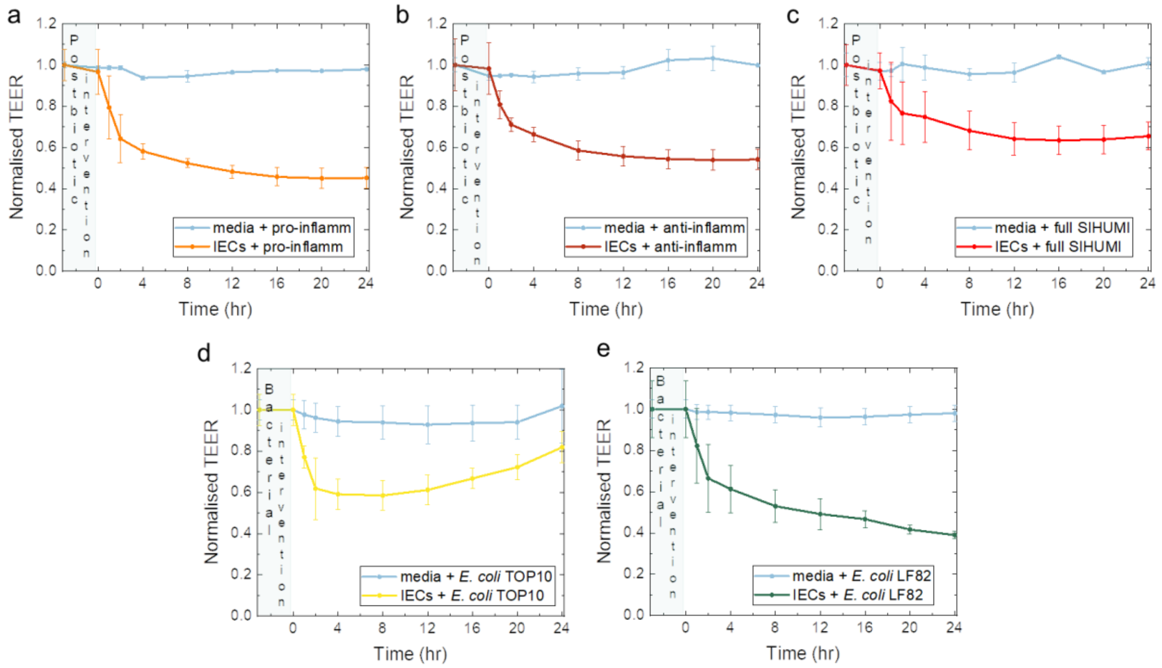

Supplementary Figure 19: Evolution of barrier integrity during the 24-hour **a-c** postbiotic and **e-f** live bacterial interventions in monolayers of IECs (3:1 Caco-2:HT29MTX cells) grown and maintained in Transwell inserts. TEER values were obtained via the software of the CellZScope automated impedance monitoring system. Each data point represents the mean ± standard deviation of one experiment, with four inserts for each point.
